# First Call Simulation: Preparing for Acute Patient Decompensation with Facilitated, Peri-Scenario Debriefing

**DOI:** 10.15766/mep_2374-8265.10982

**Published:** 2020-09-30

**Authors:** Andrew Musits, Gianna Petrone, David Lindquist, Paul George

**Affiliations:** 1 Assistant Professor, Department of Emergency Medicine, Alpert Medical School of Brown University; 2 Associate Professor, Department of Emergency Medicine, Alpert Medical School of Brown University; 3 Associate Professor, Department of Family Medicine, Alpert Medical School of Brown University; Associate Dean of Medical Education, Alpert Medical School of Brown University

**Keywords:** Simulation, Diagnostic Reasoning, Peri-Scenario Debriefing, Large-Group Simulation, Internship Preparation, Acute Care, Chest Pain, Hypotension

## Abstract

**Introduction:**

Medical students may graduate with limited experience in managing acutely decompensating patients independently. As interns they often assume that mantle of responsibility. This first call simulation-based training provided fourth-year medical students with a guided experience in creating differential diagnoses and initiating diagnostic plans while resuscitating patients.

**Methods:**

This simulation session was designed as a 3-hour session as part of a larger internship preparatory course for fourth-year medical students. The session contained three high-fidelity simulation scenarios involving a decompensating patient with a focus on developing a broad differential diagnosis and initiating treatment. The medical student leader for each patient encounter rotated with the remaining medical students actively involved in the peri-scenario debriefing process. The simulation effectiveness tool (SET-M), a published instrument to measure a learner's perception of how effective the simulation met their learning needs, was completed at the end of the session.

**Results:**

Twenty students participated in the session and completed the SET-M. Ninety-five percent of students strongly agreed they were better prepared to respond to changes in a patient's condition and felt empowered to make clinical decisions. Of students, 100% strongly agreed that the debriefing was valuable in helping to improve their clinical judgment and contributed to their learning.

**Discussion:**

Simulation-based training with facilitated, peri-scenario debriefing may be an effective method to develop confidence and clinical reasoning skills. This may help fourth-year medical students prepare for the impending responsibility to evaluate and initiate care for acutely decompensating patients.

## Educational Objectives

By the end of this activity, learners will be able to:

1. Identify potential underlying etiologies of a patient's decompensation.
2. Apply basic and advanced life support as indicated.
3. Develop an initial care plan for a decompensating patient.

## Introduction

Regardless of the specialty that graduating medical students enter, they will soon be managing inpatients that may develop urgent or emergent needs. As the covering intern, they will often be the first call or the initial provider contacted. They will be expected to evaluate the patient, make initial diagnostic and care decisions, and determine when to appropriately call for assistance.

Medical students may have limited opportunities to independently manage acutely decompensating patients during their clinical rotations. Simulation is an educational tool that allows for immediate increased responsibility without increased risk to patient safety. It has been described as an “ethical imperative” in medical education.^1^ Simulation is also useful for high-risk, low-frequency events. The importance of this educational need is recognized by the AAMC: entrustable professional activity (EPA) 10 which states, “recognize a patient requiring urgent or emergent care and initiate evaluation and management.”^2^ The Liaison Committee on Medical Education (LCME) has also recognized the importance of this topic: LCME standard 7.2 for curricular content includes the ability to “recognize and interpret symptoms and signs of disease,” and “develop differential diagnoses and treatment plans.”^3^ The development of critical judgement and problem-solving skills are also included in LCME standard 7.4.^3^

National organizations aside, if one asked medical students approaching graduation about their anxieties, they will likely include concerns about caring for acutely ill or decompensating patients.^4,5^ A simulation-based course places the responsibility on the student for the initial evaluation and treatment of the patient requiring urgent or emergent intervention. This need has been recognized by other curricula published on *MedEdPORTAL*.^6–8^

These previously published curricula were designed to recreate the experience or stress of a call shift, and teach specific medical management.^6–8^ These curricula used simulation to teach skills regarding multitasking and interruptions. The scenarios in First Call Simulation focused on the differential approach to common chief complaints, rather than the medical treatment of a specific diagnosis. This was reflected by the case titles. For example, case 3 is titled hypotension, not urosepsis. Using a facilitated process, students were challenged to create a differential diagnosis and initiate an appropriate management plan, while providing initial stabilization. The prior curricula used debriefing at the end of the simulated experience. In this course, peri-scenario debriefing used a pause and reflect debriefing technique to facilitate the learner experience and emphasize the teaching objectives.

## Methods

### Development

We implemented this 3-hour simulation session as part of the required internship preparatory course (IPC) at the Warren Alpert Medical School of Brown University. The IPC course was developed based on a recognized need of preparing students for the first day of internship, regardless of the students' chosen field. The course consisted of 26 sessions, ranging from specialty-specific reviews to procedural training and financial planning. Each session was approximately 3 hours in duration. Students chose 10 of these sessions over a 2-month period in the last semester of medical school.

We developed the scenarios for the First Call Simulation session based on EPA 10.^2^ Expert simulation faculty at our institution developed the scenarios through an iterative collaborative process. Scenarios were reviewed by operations staff for additional feedback prior to piloting with these 20 students.

### Equipment/Environment

This session consisted of three individual cases involving a decompensating patient on the general medical floors. The simulation space was constructed to mimic a typical patient room on the general medical floor. The standard set-up for all scenarios included a stretcher with a high-fidelity mannequin, telemetry monitor, crash cart, defibrillator, bag valve mask, and basic intubation equipment. Participants could order any diagnostic test, treatment, or consult they felt was appropriate. A projector screen was positioned within the space for participants to view lab results and imaging (Appendix D). Information regarding the patient's emergency department visit as well as their admission notes were built into the case. A whiteboard was available to assist the facilitator with debriefing. Chairs were positioned in the simulation room for the observing students.

### Personnel

Two faculty members (Andrew Musits and Gianna Petrone) acted as facilitators and were present for both sessions. The facilitators alternated between the roles of debriefing and acting as a nurse confederate. The medical students were divided into three groups of three to four students depending on class size. One student group was actively involved with each scenario. The remaining two groups were seated in the simulation room to observe and participate in the peri-scenario debriefing. The groups and leader for each encounter rotated, with observers actively involved in the feedback and debriefing process throughout the scenario. A simulation technician was present in the control room to ensure the equipment was functioning properly and to assist when needed.

### Implementation

The First Call Simulation session began with a 20-minute orientation to the course and simulation space. Expectations and psychological safety were discussed. Next, three to four students were selected to manage each case, with the remaining students observing in chairs located in the simulation space. The three to four students selected to actively manage the case interacted with the simulator. The remaining six to seven students in the observer seats actively participated in the peri-scenario debriefing, as detailed below. Each scenario, including the peri-scenario debriefing, lasted 40–50 minutes. The entire session, including the orientation, breaks, and time to complete feedback forms lasted 3 hours. Below is a brief overview of each case, and full simulation templates:

1. A 78-year-old was admitted for dehydration, now with altered mental status (Appendix A).
2. A 67-year-old was admitted for cellulitis, now with chest pain (Appendix B).
3. A 76-year-old was admitted for a fall with hip fracture, now with hypotension (Appendix C).

### Peri-Scenario Debriefing

One key element of this course was the use of facilitator-guided, peri-scenario debriefing. When the learners gained momentum and started placing orders for diagnostics and interventions, the facilitator would pause the scenario and asked a student in the audience for a brief recap of the events thus far. Then, turning to the learner, the facilitator might ask some advocacy inquiry questions to understand the learner's thought process. Suggestions for next steps were sought from the observers. This kept all learners engaged. This same technique of interjecting with a time-out and facilitated discussion can occur when the learners reach a point of indecision or uncertainty and required prompting or redirection. Differentials and diagnostic workup discussions occurred during the peri-scenario debriefing. Every scenario had critical actions and learning objectives to help guide the facilitator when he or she paused for peri-scenario debriefing. If the learners performed a critical action this should be highlighted. If critical actions were missed, the faculty should help the group recognize its importance so it can be accomplished when they time back into the scenario. Furthermore, faculty were trained to query learners regarding the differential diagnosis, diagnostic measures, and therapeutic measures during the debriefing.

### Assessment

This was a formative experience for the learners, and not designed to be a capstone assessment. Learners evaluated their experience in this session using the simulation effectiveness tool (SET-M). The SET-M is a published instrument to measure the learner's perception of how effective the simulation met their learning needs.^9^ We administered this feedback form electronically in a mobile-friendly format using Qualtrics. Students voluntarily completed of this feedback immediately after the session.

## Results

During the spring of academic year 2018–2019, two sessions were held with 10 fourth-year medical students attending each session for a total of 20 unique student participants. All 20 participants completed the SET-M feedback tool (100% completion) (Table). At least 95% of students strongly agreed with the following statements on the SET-M tool:

• “I am better prepared to respond to changes in my patient's condition.”
• “I felt empowered to make clinical decisions.”
• “I had the opportunity to practice my clinical decision-making skills.”
• “I am more confident in my ability to prioritize care and interventions.”
• “I am more confident in my ability to report information to the health care team.”
• “I am more confident in providing interventions that foster patient safety.”
• “Debriefing contributed to my learning.”
• “Debriefing was valuable in helping me improve my clinical judgment.”
• “Debriefing provided opportunities to self-reflect on my performance during simulation.”
• “Debriefing was a constructive evaluation of the simulation.”

**Table.**
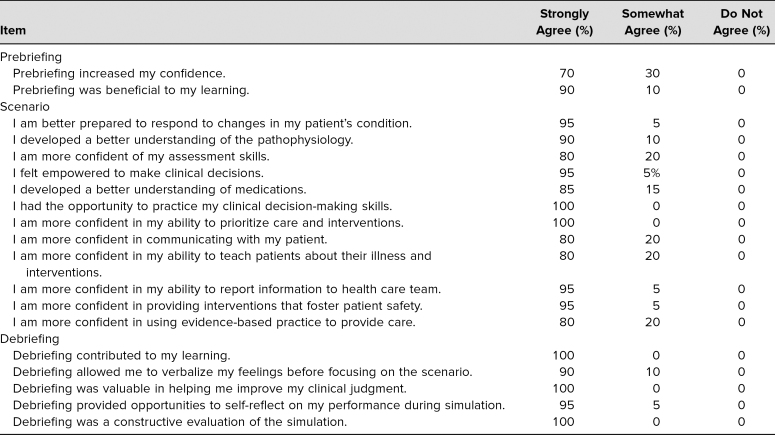
Student Responses (*n* = 20) to the Simulation Effectiveness Tool-Modified (SET-M)

Responses to the question, “What else would you like to say about today's simulated clinical experience?” focused on feeling engaged throughout the activity and having increased confidence. Common suggestions included making this available to more students.

## Discussion

The First Call Simulation differed from the existing published curricula by contributing three new simulation scenarios. Additionally, we focused this curriculum to provide formative feedback and coaching to learners as they worked through differential diagnoses and developed their clinical reasoning skills. We accomplished this with the pause and reflect teaching method described above as peri-scenario debriefing. Similar debriefing techniques have previously been described as facilitator-guided within event debriefing.^10^

Admittedly, there will be some faculty-to-faculty variation in the facilitation of this course. It was designed to be taught by clinical faculty that have the knowledge base and educational experience to make judgements and alterations as needed. However, they are guided by the learning objectives critical actions provided.

There were some limitations to the data collected. The comments listed in the results section are a representative sampling of comments from learners. However, a formal thematic analysis was not performed and was beyond the scope of this pilot project. While the SET-M has been used extensively with nursing students in the simulation lab, it must be recognized that it is subjective, self-reported data. Regardless, the authors believe it provided useful feedback pertaining to the curricular effectiveness in various domains.

Simulation is time and resource intensive.^11^ During this pilot, the group size was 10. Faculty involved agreed the teaching method could support a group of 12, allowing for three groups of four to rotate in and out of the scenarios, and keep learners engaged. Providing educational feedback is cited as the most important feature of simulation-based education.^12^ Effectively performing facilitator guided peri-scenario debriefing was critical to the success of this session. It required an experienced faculty member comfortable with simulation as a teaching modality. While additional faculty could be recruited to help teach future sessions, they would likely benefit from formal debriefing training and mentorship. Based on student feedback and demand, we decided to double the number of sessions for academic year 2019–2020. Based on faculty feedback, class enrollment was also increased from 10 to 12 students per session.

Based on student feedback and our analysis of the SET-M responses, we also better understood the skills taught in this course. Skills such as communication, patient assessment, and evidence-based medicine were noted to be the weakest areas of this session based on the SET-M data (the items with only 80% strong agreement). We suspect this was due to the learning modality used. That is, talking to a high-tech mannequin is not the best educational approach for communication skills. Rather, role-play or standardized patient encounters may better accomplish communication-based objectives.

Fortunately, the items on the SET-M that corresponded to our objectives were rated more highly by the students. The Set-M statement, “I developed a better understanding of the pathophysiology” supported curriculum Objective 1. “I had the opportunity to practice my clinical decision-making skills” and “I am more confident in my ability to prioritize care and interventions” supported Objective 2. “I am better prepared to respond to changes in my patient's condition” and “I felt empowered to make to make clinical decisions” supported Objective 3.

Emergency medicine faculty taught this course. The clinical skills of these faculty aligned with the course objectives, as clinical decision-making and initial resuscitation of patients formed the core of emergency medicine practice. However, this course could be taught by faculty who work in any acute or inpatient setting.

This course was not specialty-specific and was designed to be useful to all students regardless of their chosen specialty. The patient presentations were general and could be encountered in the emergency department or any medical-surgical ward, regardless of whether the patient was admitted to surgery, oncology, medicine, or another subspecialty.

The skills taught in this course were chosen to improve the preparation of senior medical students to provide care for patients in diverse settings. This may alleviate some anxiety as learners graduate medical school and begin internships better positioned to manage acutely decompensating patients.

## Appendices

Altered Mental Status Simulation.docxChest Pain Simulation.docxHypotension Simulation.docxCase Images.pptx
All appendices are peer reviewed as integral parts of the Original Publication.
