# Supplementary material for: First Call Simulation: Preparing for Acute Patient Decompensation with Facilitated, Peri-Scenario Debriefing
Source: MedEdPORTAL. 2020 Sep 30:10982. doi: 10.15766/mep_2374-8265.10982 (PMC7526501; doi:10.15766/mep_2374-8265.10982)
Supplement: Supplementary file 1 — Altered Mental Status Simulation.docxChest Pain Simulation.docxHypotension Simulation.docxCase Images.pptx [file mep_2374-8265.10982-s001.zip › A. Altered Mental Status Simulation.docx]

| **SIMULATION CASE TITLE: Altered Mental Status – First Call**  **Author: Andrew Musits MD, Gianna Petrone DO**  **Learner Audience: Senior Medical Students** | |
| --- | --- |
| **PATIENT NAME: Dominick Dombrowski**  **PATIENT AGE: 78 years**  **CHIEF COMPLAINT: Admitted for dehydration, now c/o altered mental status.**  **PHYSICAL SETTING: Med-Surg ward** | |
| **Brief narrative description of case** | The patient is admitted to the medical service for dehydration secondary to a GI illness. He presented to the hospital with 3 days of non-bloody vomiting and some diarrhea. Abd/pelvis CT scan in the ED yesterday showed no acute pathology, mild bowel wall thickening. Admitted for IVF due to intractable vomiting. The learner is currently covering on night float, and receives a call that the patient is found by the nurse, more somnolent and difficult to arouse, but protecting his airway. The learner needs to work through a timely differential to evaluate for emergent conditions. The head CT will show an intraparenchymal ICH, and the patient’s care will need to be escalated appropriately. |
| **Learning Objectives** | 1. Discuss potential diagnosis of the patient’s newly developed altered mental status. 2. Recognition of acute ICH on head CT. 3. Demonstrates appropriate management of an inpatient with newly diagnosed ICH. |
| **Critical Actions** | - Obtain a focused history from RN - Perform a focused physical exam - Obtain a STAT fingerstick glucose - Review medications, consider Narcan - Order labs (may include CBC, CMP, NH4, Cultures) - Obtain urinalysis - Administer oxygen - Order CT head - Raise head of the bed - Reverse coumadin - Reduce blood pressure - Call for help |

| Initial Presentation | | | |
| --- | --- | --- | --- |
| **Initial vital signs** | HR:88, RR:12, BP:188/98, O2: 95%, Temp: 97.2 | | |
| **Overall Appearance** | 78-year-old male, somnolent | | |
| **Actors and roles in the room at case start** | The patient is a high-technology mannequin. | | |
| **HPI** | The patient is admitted to the medical service for dehydration secondary to a GI illness. He presented to the hospital with 3 days of non-bloody vomiting and some diarrhea. Abd/pelvis CT scan in the ED yesterday showed no acute pathology, mild bowel wall thickening. Admitted for IVF due to intractable vomiting. The RN calls you because the patient is more somnolent and difficult to arouse. He has been alert all day, only a 1 person assist to the commode. The last time the RN saw the patient was 2 hours ago, when vital signed were checked. Additional history cannot be obtained due to altered mental status. | | |
| **Past Medical/Surgical History** | **Medications** | **Allergies** | **Family/social History** |
| Hypertension  A Fib  Hyperlipidemia | Metoprolol  Coumadin  Simvastatin | NKDA | Lives independently  1/2 PPD smoker  No Alcohol  No Drugs |
| **Physical Examination** | | | |
| General – Laying in hospital bed. Appears to be sleeping.  Head – Atraumatic  Eyes – Pupils 2mm equal and sluggish  ENT – Normal  Neck - Normal  Cardiovascular – RRR. No murmurs. Symmetric radial pulses.  Resp – No upper airway noises or gargling. Good air entry b/l without wheezing. No increased work of breathing.  Skin – Normal  Abdomen – Normal  Extremities - Normal  Musculo Skeletal – Normal  Neurological – Somnolent. Face symmetric. Opens eyes spontaneously. Responds to brisk stimuli. Mainly moaning and groaning. May verbalize “Don’t feel well” or “Leave me alone” Moves all four extremities with a purposeful response to pain. | | | |

| Scenario Triggers and Progression | | |
| --- | --- | --- |
| **Intervention/ Time point** | **Change in case** | **Additional information** |
| **State 1: Initial Presentation**  Rhythm: Sinus  HR: 88/min  BP: 188/98  RR: 12  O_2_SAT: 98 %  T: 97.2^o^F | History and Physical  Vital Signs  Monitor  Oxygen  Fingerstick  Med review / give naloxone  Order labs  Order EKG  Order head CT | No fingerstick in 5 mins -> RN prompt: “Is he a diabetic?”  No med review / naloxone / EKG in 5 min -> RN prompt: “Do you think he overdosed on something?”  No CT in 8 min -> RN prompt: “Do you want any imaging doc?”  No head CT in 10 min -> State 3  Head CT resulted -> State 2 |
| **State 2: Head CT resulted**  Rhythm: Sinus  HR: 88/min  BP: 188/98  RR: 12  O_2_SAT: 98 % | Call senior  Page Neurosurgery  Raise head of the bed  Reverse coumadin  Control BP | Call to senior -> Will ask about blood pressure and anticoagulation.  PCC + BP control + Call to Neurosurgery -> End Case |
| **State 3: Decompensation/Seizure**  Rhythm: Sinus Tach  HR: 120/min  BP: 200/102  RR: 8  O_2_SAT: 93 % | Benzodiazepines  Airway maneuvers  RN will call Code in 30 seconds if not requested by learner | Benzodiazepine ->No response  Calls Code -> Senior Arrives -> Patient intubated -> CT scan -> State 2 |

**Ideal Scenario Flow**

The learners enter the room to find a patient with altered mental status, unable to provide much history. A physical exam, including a neurologic exam will be performed. The learners will consider a broad differential including hypoglycemia (check a fingerstick) and opiate overdose (check pupils or give naloxone.) When no immediate cause of the altered mental status is found, the learns will order a broad panel of labs, urinalysis, chest x-ray, and head CT. While awaiting the results of the head CT, it is usually effective to time out and review the learner’s differential, therapeutic and diagnostic actions. Based on timing the, skill level of the group, the facilitator may choose to enter state 3 prior to the head CT resulting. If this occurs, the learner will administer benzodiazepines and protect the patient’s airway. After the head CT results the emergent nature of the patient’s condition and CT will be communicated to the senior resident, attending, or neurosurgeon on call. The learners will initiate basic treatments such as elevating the head of the bed, reversing warfarin, and controlling the blood pressure.

**Anticipated Management Mistakes**

1. Omission of a neurologic exam: Some learners will fail to perform a neurologic exam because the patient is altered. This can be addressed during the first time out, when differentials are being discussed. The facilitator might ask the observers “are their other neurologic exam findings you would like to know in this patient” to prompt additional suggestions and relate them to the working differential.
2. Failure to consider a broad differential diagnosis: Some learners will have premature closure, and focus on a single diagnostic possibility before any of the tests return. During the time out, their peers will often offer a broad list of potential diagnoses, highlighting this area in which they can improve.
3. Failure to recognize treatments for ICH prior to neurosurgical intervention: After obtaining the head CT, learners will reflexively call neurosurgery but may fail to perform other interventions such as blood pressure control and reversal of warfarin. This can be addressed with a time out and facilitated discussion with the group, or prompts can be provided by the neurosurgical consultant.

| Supporting Documents and media | |
| --- | --- |
| **Labs** | Basic admit labs. Mild Lactate, some heme concentration, mild AKI, normal K |
| **EKG** | **EKG: NSR** |
| **Imaging** | **Head CT: Intraparenchymal Bleed**  **CXR: Pre and Post Intubation** |
| **Other** |  |

ECG 1: ^1^


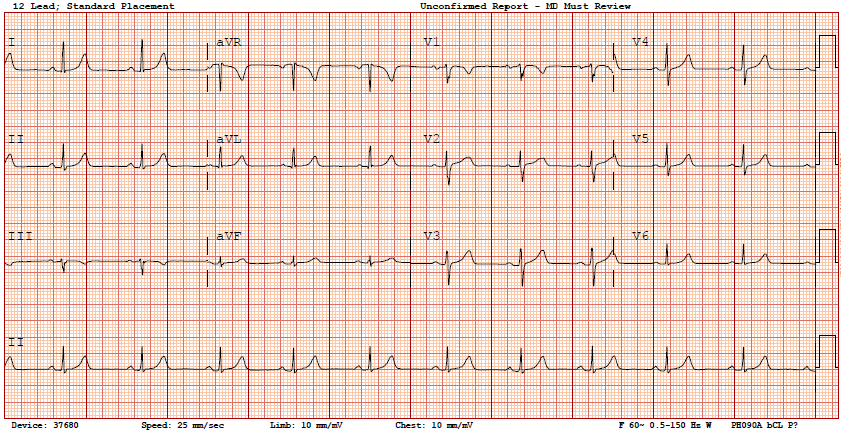


Post Intubation^1^


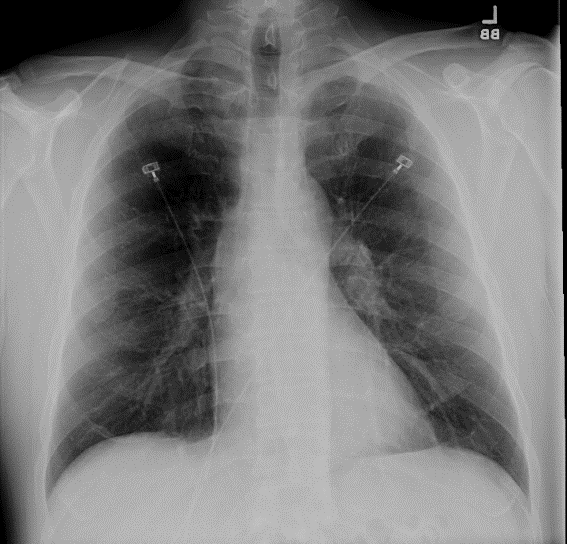


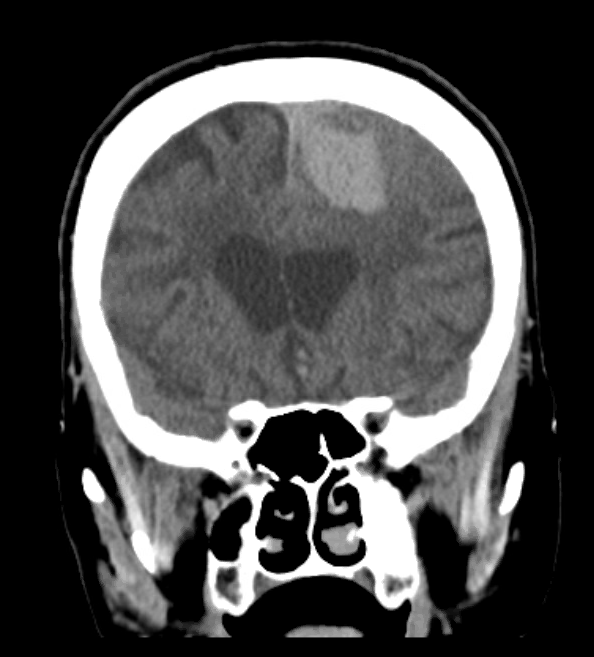
CT Brain: ^1^


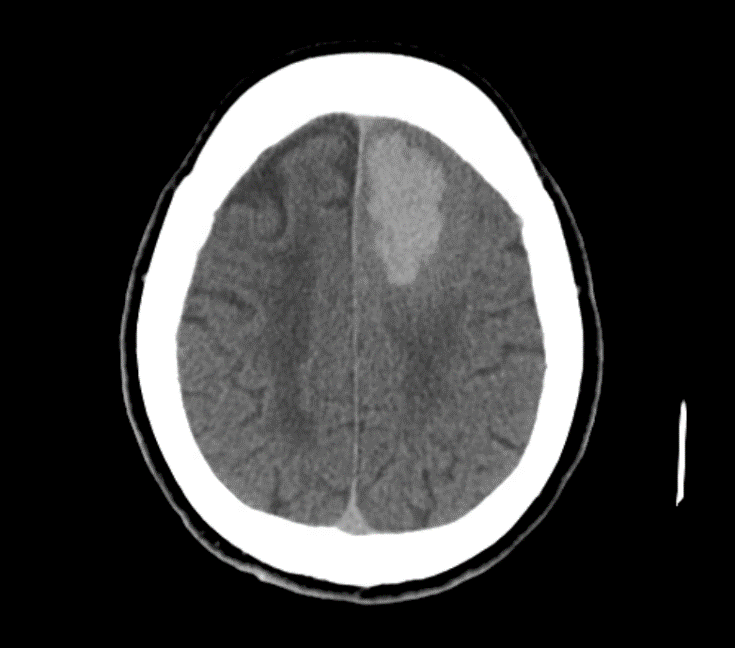


| **Basic Metabolic Panel** | |
| --- | --- |
| Glucose | 110 |
| BUN | 20 |
| Creatinine, Ser | 1.8 |
| Sodium | 132 |
| Potassium | 3.6 |
| Chloride | 100 |
| CO2 | 20 |
| Anion Gap |  |
| Calcium |  |
| eGFR |  |

| **CBC with Diff** | |
| --- | --- |
| WBC | 4 |
| RBC |  |
| Hemoglobin | 16 |
| Hematocrit | 48 |
| MCV |  |
| MCH |  |
| MCHC |  |
| RDW |  |
| Platelets | 180 |

| **Venous Gas** | |
| --- | --- |
| pH | 7.32 |
| PCO2 | 40 |
| PO2 | 80 |
| HCO3 | 20 |
| Potassium | 3.5 |
| Lactate | 2.0 |

| **Urine** | |
| --- | --- |
| Color | clear |
| pH | 6 |
| Prot | Neg |
| Glu | Neg |
| Spec Gr | 1.02 |
| Ketones | 0 |
| Nitrites | 0 |
| RBC | 0 |
| WBC | 2 |
| Bacteria | none |
| Squams | Few |

Image Citations:

1) Author Owned
