# Supplementary material for: First Call Simulation: Preparing for Acute Patient Decompensation with Facilitated, Peri-Scenario Debriefing
Source: MedEdPORTAL. 2020 Sep 30:10982. doi: 10.15766/mep_2374-8265.10982 (PMC7526501; doi:10.15766/mep_2374-8265.10982)
Supplement: Supplementary file 1 — Altered Mental Status Simulation.docxChest Pain Simulation.docxHypotension Simulation.docxCase Images.pptx [file mep_2374-8265.10982-s001.zip › B. Chest Pain Simulation.docx]

| **SIMULATION CASE TITLE: Chest Pain – First Call**  **Author: Andrew Musits MD, Gianna Petrone DO**  **Learner Audience: Senior Medical Students** | |
| --- | --- |
| **PATIENT NAME: Jeremy Jones**  **PATIENT AGE: 67 years**  **CHIEF COMPLAINT: Admitted for Cellulitis, now c/o chest pain.**  **PHYSICAL SETTING: Med-Surg ward** | |
| **Brief narrative description of case** | The patient is admitted to the medical service for cellulitis of the left arm, refractory to outpatient antibiotics. He is non-insulin dependent diabetic. The learner is currently covering on night float, and receives a call that the patient is complaining of chest pain. The learner needs to work through a timely differential to evaluate for emergent conditions. The EKG will reveal a STEMI, and the patient’s care will need to be escalated appropriately. |
| **Learning Objectives** | 1. Discuss potential diagnosis of the patient’s newly developed chest pain. 2. Recognition of STEMI on EKG 3. Demonstrates appropriate management of an inpatient with newly diagnosed STEMI |
| **Critical Actions** | - Obtain a focused history - Perform a focused physical exam - Obtain a STAT EKG and Chest X-ray - Recognition of STEMI - Administer 324 mg of ASA - Treat pain with SL nitroglycerine followed by morphine - Administer oxygen for O2 sat <92% - Call for help / activate interventional cardiology according to local protocols - Explain the working diagnosis to the patient |

| Initial Presentation | | | |
| --- | --- | --- | --- |
| **Initial vital signs** | HR:92, RR:18, BP:142/88, 98 O2 %, Temp: 97.2 | | |
| **Overall Appearance** | 67-year-old clutching his chest. | | |
| **Actors and roles in the room at case start** | The patient can be a high-technology mannequin or standardized patient. | | |
| **HPI** | The patient is admitted to the medical service for cellulitis to the left arm, refractory to outpatient antibiotics. The RN called you because the patient is complaining of chest pain. The pain began after he walked his family down to the lobby after their visit this evening. The pain is constant, 8/10, described as a pressure in his chest. It hurts when he breathes. No cough. No hemoptysis. He his nauseas, no vomiting. No trauma. He has never had pain like this before. The cellulitis has been improving, no fevers. | | |
| **Past Medical/Surgical History** | **Medications** | **Allergies** | **Family/social History** |
| Diabetes  Hypertension | Clindamycin  Lisinopril | PCN – Rash | Unknown |
| **Physical Examination** | | | |
| General – Sitting semi-recumbent. Clutching his chest. Holding an emesis basin.  Head – Atraumatic  Eyes – Normal  ENT – Normal  Neck - Normal  Cardiovascular – RRR. No murmurs. Symmetric radial pulses.  Lungs – No wheezing. Slight crackles in the b/l bases. No increased work of breathing.  Skin – Warm, diaphoretic. Erythema to the Left arm with-out subcutaneous air.  Abdomen – Normal  Extremities - No edema, full ROM.  Musculo Skeletal – Normal. No reproducible chest pain on palpation.  Neurological – A&O x3 | | | |

| Scenario Triggers and Progression | | |
| --- | --- | --- |
| **Intervention / Time point** | **Change in case** | **Additional information** |
| **State 1: Initial Presentation**  Rhythm: Sinus Tach  HR: 92/min  BP: 142/88  RR: 18  O_2_SAT: 98 %  T: 97.2^o^F | History and Physical  Vital Signs  Monitor  Oxygen  Order CXR  Order EKG | Learner obtains vitals and orders EKG -> State 2  No EKG available until learner listens to heart/lungs and obtains vitals.  If no EKG in 6 min: Prompt from patient “You don’t think this is my heart, doc?” |
| **State 2: EKG Results**  Rhythm: Sinus Tach  HR: 92/min  BP: 142/88  RR: 18  O_2_SAT: 98 % | Recognition of STEMI on EKG  If STEMI not recognized, prompt from RN: “What do you make of that EKG?” Or “That EKG doesn’t look good to me, want to call your senior?” | ASA and SL Nitro -> State 3  No appropriate action/no call for help in 6 min -> State 4 |
| **State 3: Pain improved**  Rhythm: Sinus Tach  HR: 88/min  BP: 108/70  RR: 18  O_2_SAT: 98 % | Call to senior and/or cardiology -> Advise learner to bolus heparin and prepare patient for transport to cath lab | Call to senior -> END case  Fail to call for help -> State 4 |
| **State 4: Decompensation**  Rhythm: V Fib  HR: --  BP: --  RR: --  O_2_SAT: 88 % | CPR  Defibrillate  BVM ventilation  ACLS Meds  Call for help | Defibrillation -> State 3 |

**Ideal Scenario Flow**

The learners enter the room to find a patient with chest pain. They will perform a history that is concerning for an acute coronary syndrome. The physical is unremarkable for acute abnormalities. Learners will typically order a chest x-ray and EKG, along with a panel of labs including troponin. While results are pending, we suggest timing out of the scenario to discuss the working differential diagnoses. Upon re-entering the scenario, the chest x-ray and EKG (STEMI) are available. The learners will recognize the EKG abnormality as a time sensitive emergency, initiate treatment, including aspirin and nitroglycerin. They will then call their senior resident, attending, or interventional cardiologist on call. They will clearly state their concern for the patient with a STEMI and active chest pain, and develop a plan to move emergently to the cardiac catheterization lab. Based on timing, and the maturity/skill of the learners, the facilitator may choose to end the scenario at that point, or enter State 4.

**Anticipated Management Mistakes**

1. Omission of the physical exam: Some learners will fail to perform a physical exam. This can best be addressed during the first time out, when differential diagnoses are being discussed. They will often recognize physical exam findings (symmetric lung sounds, peripheral pulses) can help guide their differential.
2. Failure to consider a broad differential diagnosis: Some learners will have premature closure, and focus on a single diagnostic possibility before any of the tests return. During the time out, their peers will often offer a broad list of potential diagnoses, highlighting this area in which they can improve.
3. Uncertainty about who to call once a STEMI is recognized: This may vary based on the practice environment, and can be addressed during the debriefing. The key is that learners recognize the EKG changes as a time sensitive emergency.
4. Failure to prepare for transport to cardiac catheterization lab: Learners may not appreciate the potential for this patient to decompensate to V-fib while awaiting intervention. If the facilitator chooses to enter state 4 after the call with cardiology, a discussion about preparing for decompensation (ensuring IV access, placing defibrillator pads) can follow. If the learners do not respond to V-Fib in a timely fashion, the scenario can be re-wound after a time out and debriefing to allow them to end on success.

| Supporting Documents and media | |
| --- | --- |
| **Labs** | Admission labs are available with mild Leukocytosis |
| **EKG** | **STEMI** |
| **Imaging** | **CXR: Normal** |
| **Other** |  |





EKG: STEMI^1^

CXR: ^1^


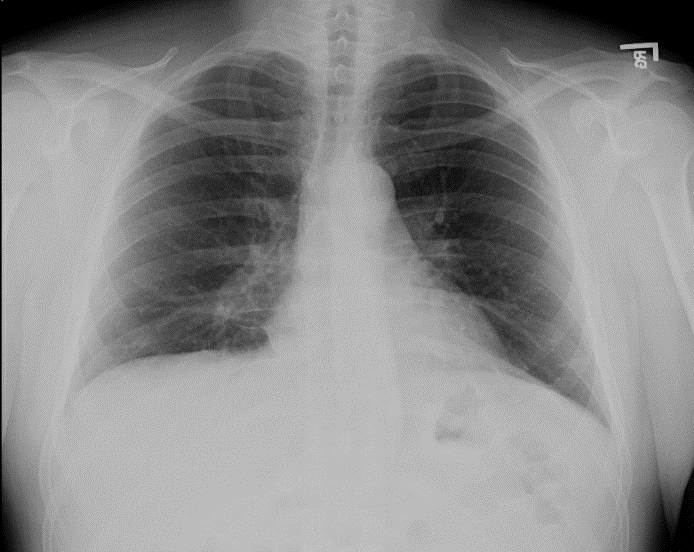


| **Basic Metabolic Panel** | |
| --- | --- |
| Glucose | 110 |
| BUN | 20 |
| Creatinine, Ser | 1.2 |
| Sodium | 138 |
| Potassium | 3.6 |
| Chloride | 100 |
| CO2 | 20 |
| Anion Gap |  |
| Calcium |  |
| eGFR |  |

| **CBC with Diff** | |
| --- | --- |
| WBC | 12 |
| RBC |  |
| Hemoglobin | 12 |
| Hematocrit | 36 |
| MCV |  |
| MCH |  |
| MCHC |  |
| RDW |  |
| Platelets | 180 |

| **Venous Gas** | |
| --- | --- |
| pH | 7.30 |
| PCO2 | 40 |
| PO2 | 80 |
| HCO3 | 20 |
| Potassium | 3.5 |
| Lactate | 2.0 |

| **Urine** | |
| --- | --- |
| Color | clear |
| pH | 6 |
| Prot | Neg |
| Glu | Neg |
| Spec Gr | 1.02 |
| Ketones | 0 |
| Nitrites | 0 |
| RBC | 0 |
| WBC | 2 |
| Bacteria | none |
| Squams | Few |

Image Citations:

1) Author owned
