# Supplementary material for: First Call Simulation: Preparing for Acute Patient Decompensation with Facilitated, Peri-Scenario Debriefing
Source: MedEdPORTAL. 2020 Sep 30:10982. doi: 10.15766/mep_2374-8265.10982 (PMC7526501; doi:10.15766/mep_2374-8265.10982)
Supplement: Supplementary file 1 — Altered Mental Status Simulation.docxChest Pain Simulation.docxHypotension Simulation.docxCase Images.pptx [file mep_2374-8265.10982-s001.zip › C. Hypotension Simulation.docx]

| **SIMULATION CASE TITLE: Hypotension – First Call**  **Author: Andrew Musits MD, Gianna Petrone DO**  **Learner Audience: Senior Medical Students** | |
| --- | --- |
| **PATIENT NAME: Susan Sweeney**  **PATIENT AGE: 76 years**  **CHIEF COMPLAINT: Admitted for fall with hip fracture, now c/o hypotension.**  **PHYSICAL SETTING: Med-Surg ward** | |
| **Brief narrative description of case** | The patient is admitted to the medical service for a femoral neck fracture, awaiting surgery by orthopedics tomorrow. The learner is currently covering on night float, and receives a call that the patient has low blood pressure on routine vitals. The learner needs to work through a timely differential to evaluate for emergent conditions. The patient will be notably febrile, tachycardic, and hypotensive. Urinalysis from the time of admission will be consistent with urinary tract infection. The patient will initially respond to fluids, but will become refractory if antibiotics are not given for sepsis. |
| **Learning Objectives** | 1. Develop a working differential for the patient with hypotension 2. Recognize signs of sepsis 3. Treat sepsis with appropriate fluid resuscitation and early antibiotics |
| **Critical Actions** | - Obtain a focused history - Perform a focused physical exam - Obtain a temperature - Initiate IV fluid bolus for hypotension - Evaluate for arrhythmia on EKG - Consider acute blood loss (Abdominal Exam, thigh exam, Rectal exam for GI bleed) - Once triad of fever, tachycardia, and hypotension are recognized, begin broad spectrum antibiotics - Call for help / activate senior resident or MICU consult - Explain the working diagnosis to the patient |

| Initial Presentation | | | |
| --- | --- | --- | --- |
| **Initial vital signs** | HR:124, RR:18, BP:88/42, 96 O2%, Temp: 101.6 | | |
| **Overall Appearance** | 76-year-old in bed, felling generally weak but awake without specific complaints. | | |
| **Actors and roles in the room at case start** | The patient can be a high-technology mannequin or standardized patient. | | |
| **HPI** | The patient is admitted to the medical service for a femoral neck fracture, awaiting surgery by orthopedics tomorrow. You receive a call from the RN that the patient has low blood pressure on routine vitals. The patient feels generally weak. In fact, she has been generally weak for several days, and thinks that is why she fell. No vomiting. No diarrhea. No bloody or melanotic stools. Denes headache, chest pain, back pain or abdominal pain. She will endorse frequent urination if asked.  The patient has no pain, received a femoral nerve block in the ED. The admitting nurse placed a urinary catheter. | | |
| **Past Medical/Surgical History** | **Medications** | **Allergies** | **Family/social History** |
| Coronary Artery Disease  PCI w/ stent – 10 years ago  Hypertension  Hyperlipidemia  Cholecystectomy- 30 years ago | Lisinopril  Metoprolol  ASA  Clopidogrel  Pravastatin | Sulfa | Unknown |
| **Physical Examination** | | | |
| General – Sitting semi-recumbent.  Eyes – Normal  ENT – Mucus membranes tacky  Neck - Normal  Cardiovascular – Tachycardic. No murmurs. Strong DP pulses bilaterally.  Lungs – No wheezing. Lungs CTA b/l. No increased work of breathing.  Skin – Warm, dry.  Abdomen – Normal  Extremities – Soft compartments to the thigh. No swelling. No edema, no sensation or movement LLL, otherwise full ROM x3.  Musculo Skeletal – Normal.  Neurological – A&O x3 | | | |

| Scenario Triggers and Progression | | |
| --- | --- | --- |
| **Intervention /Time point** | **Change in case** | **Additional information** |
| **State 1: Initial Presentation**  Rhythm: Sinus tach  HR: 124/min  BP: 88/42  RR: 18  O_2_SAT: 96 %  T: 101.6^o^F | History and Physical  Vital Signs  Obtain temperature  Monitor  Med review  Inpatient lab review  Order EKG  Bolus IVF  Tylenol  Rectal Exam | Bolus 1 L -> State 2  If no temp obtained in 6 min-> Patient will add “I feel like I have the chills”  Rectal exam-> No blood  No IVF in 6 min -> State 4 |
| **State 2: Mild improvement**  Rhythm: Sinus tach  HR: 108/min  BP: 96/52  RR: 18  O_2_SAT: 96 %  T: 101.6^o^F | Continue IVF  Broad spectrum antibiotics | If no record review in 8 minutes -> Prompt from RN “Did you want to see any of the admission labs?”  Appropriate antibiotics -> State 3  No antibiotics in 8 min -> State 4 |
| **State 3: Sepsis treatment**  Rhythm: Sinus  HR: 96/min  BP: 106/58  RR: 18  O_2_SAT: 96 %  T: 100.2^o^F | Call senior / MICU  Discuss diagnosis with patient | END CASE |
| **State 4: Decompensation**  Rhythm: Sinus tach  HR: 132/min  BP: 72/34  RR: 18  O_2_SAT: 96 %  T: 101.6^o^F | Continue IVF  Antibiotics  Vasopressors | May receive prompts from RN: “Look at that temp” or “Could this be sepsis?”  IVF + Antibiotics + Vasopressor ->State 3 |

**Ideal Scenario Flow**

The learners will enter the room to find the patient without specific complaints. They will obtain vital signs and note that in addition to the reported hypotension, the patient is tachycardic. They will perform a physical exam and history. With the combination of tachycardia and hypotension, the learners will initiate IV fluids. They will order diagnostic tests, often a panel of labs including a lactate, urinalysis, chest x ray, and EKG. Due to limited specific information, learners may find this to be a stuck point. A time out can allow for discussion of working differentials, diagnostic and therapeutic actions. Once the learners obtain a temperature, the triad of fever, tachycardia and hypotension will trigger administration of broad-spectrum antibiotics. The case ends by transferring this septic patient to the ICU.

**Anticipated Management Mistakes**

1. Delay in resuscitation with IV fluids: We found many learners to be hesitant to begin treatment prior to confirming a diagnosis. To address this during the time out, we found it helpful to ask students to divide their actions into diagnostic and therapeutic categories. Often all of their actions would be purely diagnostic, and this would lead to a discussion about what can be done to treat the patient while awaiting diagnostic results. We also found this to be a productive time to talk about uncertainty in medicine.
2. Failure to obtain a temperature: We found most learners requested or obtained vital signs including heart rate, blood pressure, and oxygen saturation, but failed to obtain a temperature. When the differential diagnosis is discussed during the time out, most students will recognize how helpful this piece of information is.
3. Failure to consider a broad differential diagnosis: Some learners will have premature closure and focus on a single diagnostic possibility before any of the tests return. During the time out, their peers will often offer a broad list of potential diagnoses, highlighting this area in which they can improve.

| Supporting Documents and media | |
| --- | --- |
| **Labs** | Basic admit labs with mild leukocytosis, elevated lactate, Urine with WBCs and Nitrites |
| **EKG** | **Sinus Tach** |
| **Imaging** | **CXR normal, Pelvis with hip fx**  **CT head, CT abd no images, but reported at “normal”** |
| **Other** |  |

EKG^1^


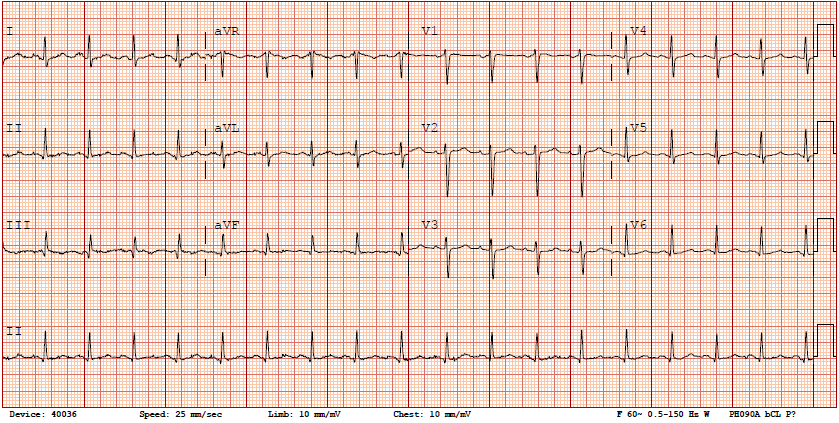


X rays: ^1^


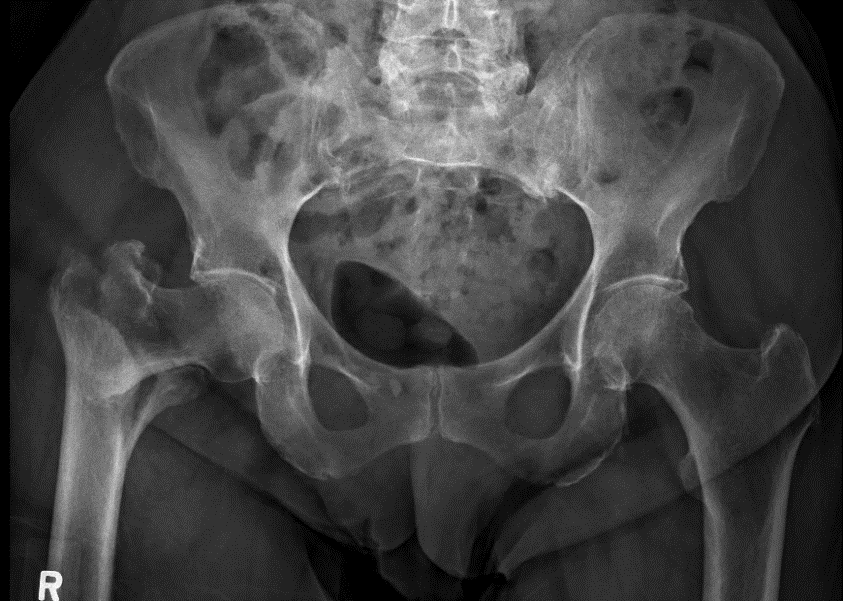


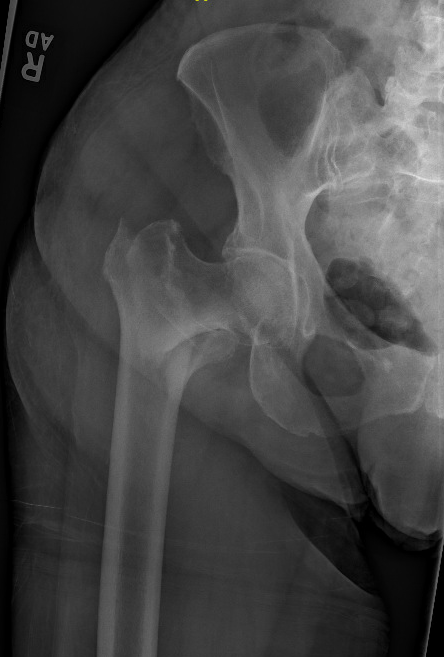

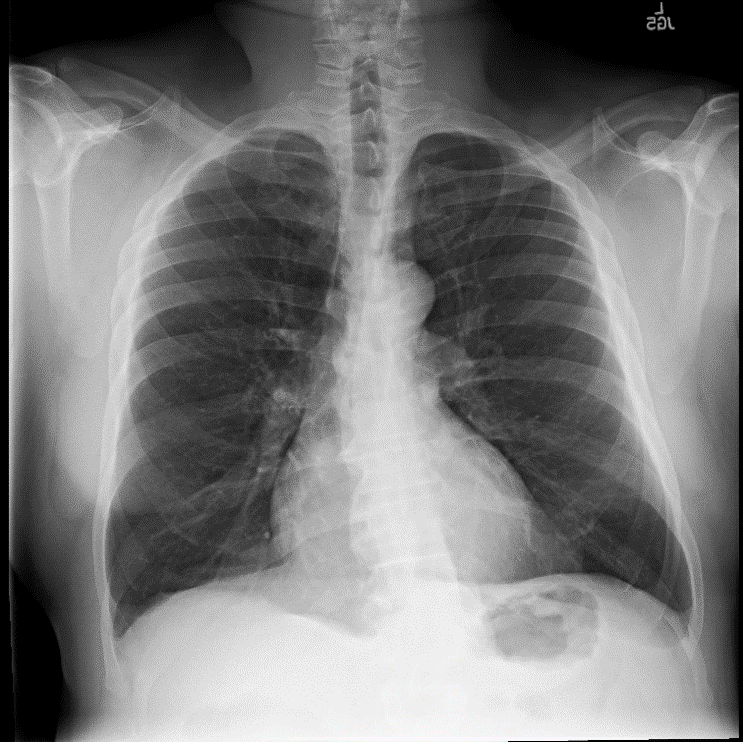


| **Basic Metabolic Panel** | |
| --- | --- |
| Glucose | 110 |
| BUN | 20 |
| Creatinine, Ser | 1.2 |
| Sodium | 132 |
| Potassium | 3.6 |
| Chloride | 100 |
| CO2 | 15 |
| Anion Gap |  |
| Calcium |  |
| eGFR |  |

| **CBC with Diff** | |
| --- | --- |
| WBC | 14 |
| RBC |  |
| Hemoglobin | 12 |
| Hematocrit | 36 |
| MCV |  |
| MCH |  |
| MCHC |  |
| RDW |  |
| Platelets | 180 |

| **Venous Gas** | |
| --- | --- |
| pH | 7.21 |
| PCO2 | 38 |
| PO2 | 80 |
| HCO3 | 15 |
| Potassium | 3.5 |
| Lactate | 2.0 |

| **Urine** | |
| --- | --- |
| Color | Cloudy |
| pH | 6 |
| Prot | 8 |
| Glu | neg |
| Ketones | 0 |
| Nitrites | Positive |
| RBC | +1 |
| WBC | >100 |
| Bacteria | many |
| Squams | <1 |

Image Citations:

1) Author owned
