# Supplementary material for: First Call Simulation: Preparing for Acute Patient Decompensation with Facilitated, Peri-Scenario Debriefing
Source: MedEdPORTAL. 2020 Sep 30:10982. doi: 10.15766/mep_2374-8265.10982 (PMC7526501; doi:10.15766/mep_2374-8265.10982)
Supplement: Supplementary file 1 — Altered Mental Status Simulation.docxChest Pain Simulation.docxHypotension Simulation.docxCase Images.pptx [file mep_2374-8265.10982-s001.zip › D. Case Images.pptx]

## Slide 1
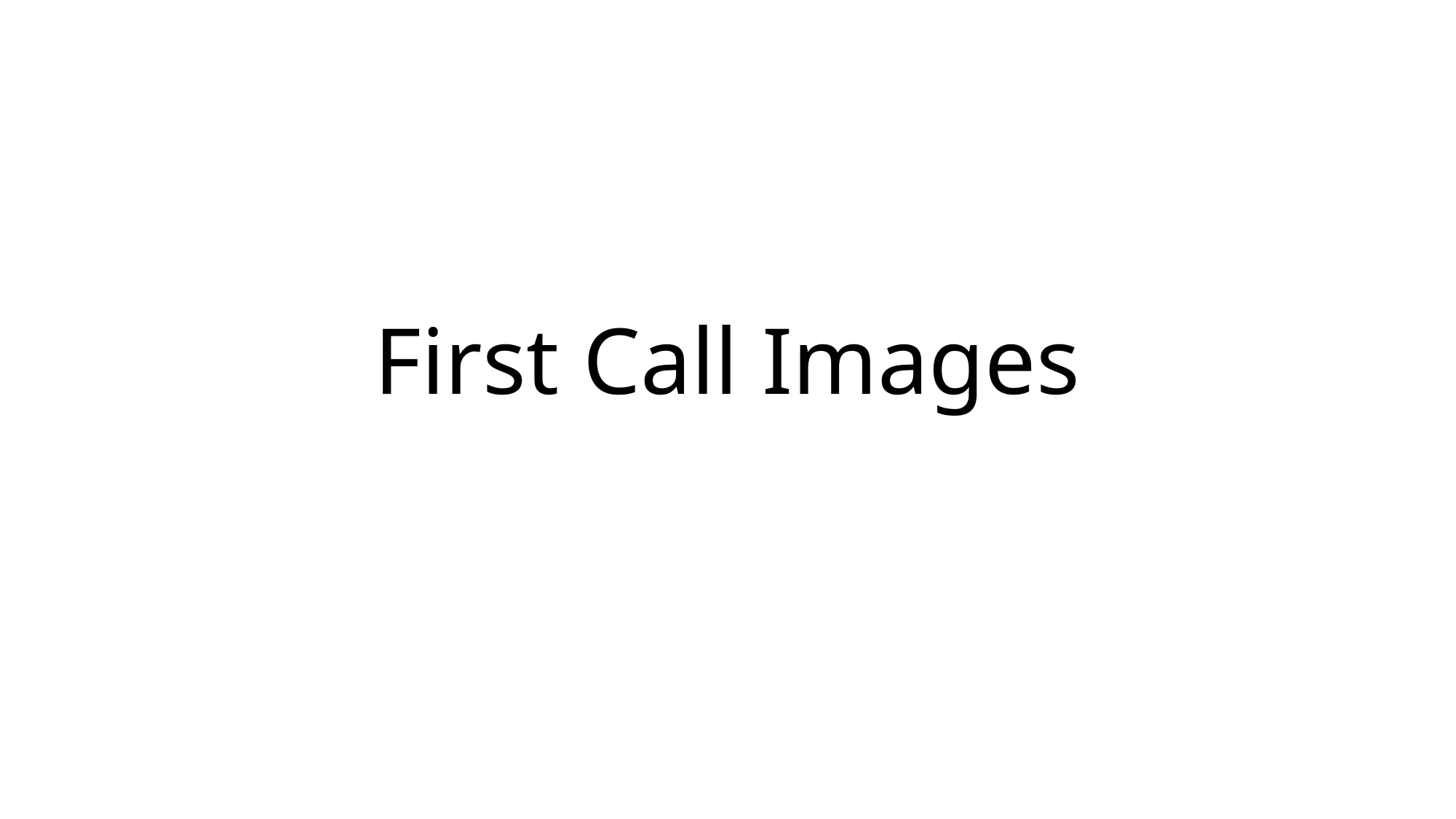

# First Call Images

## Slide 2
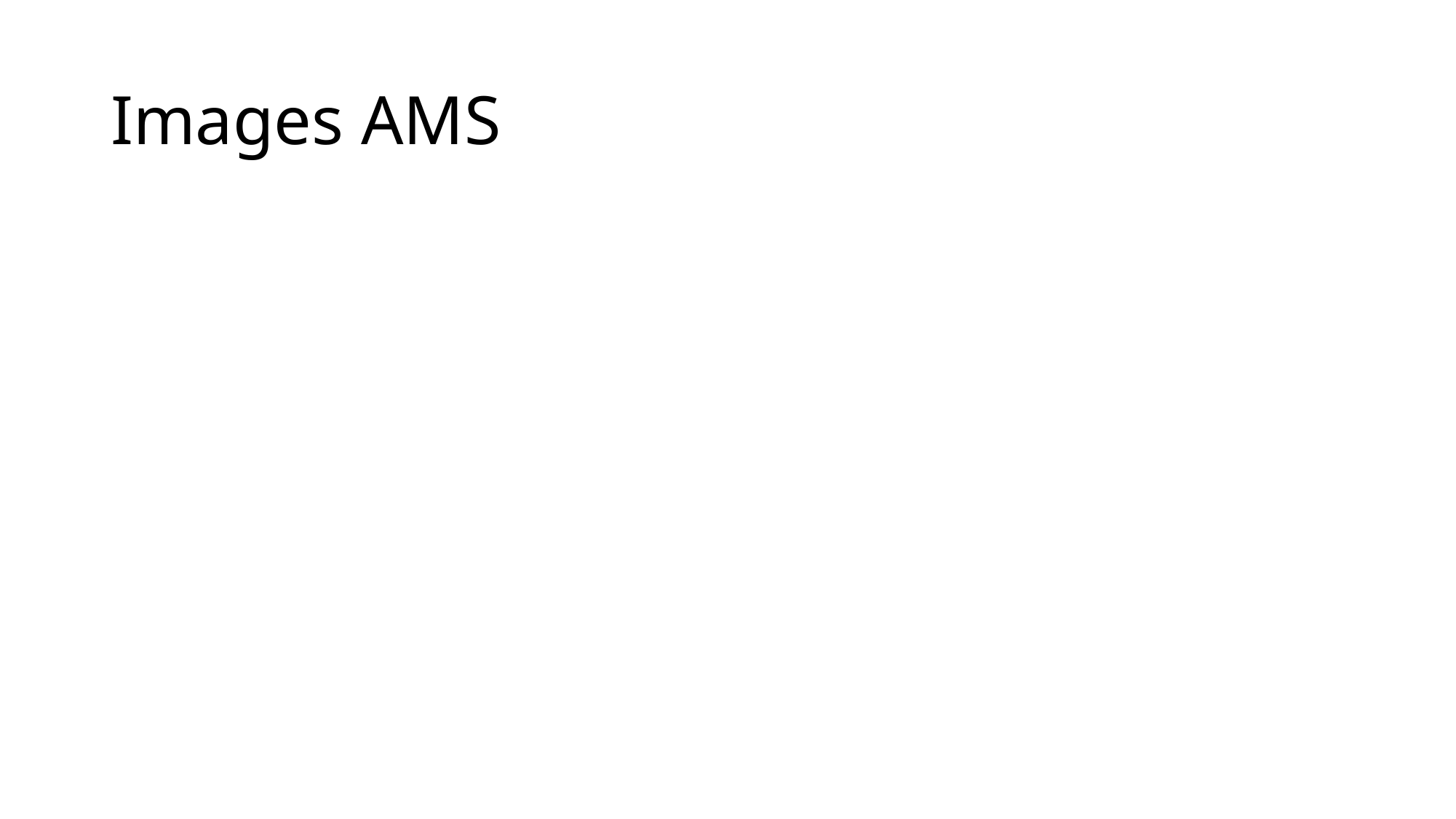

# Images AMS

## Slide 3
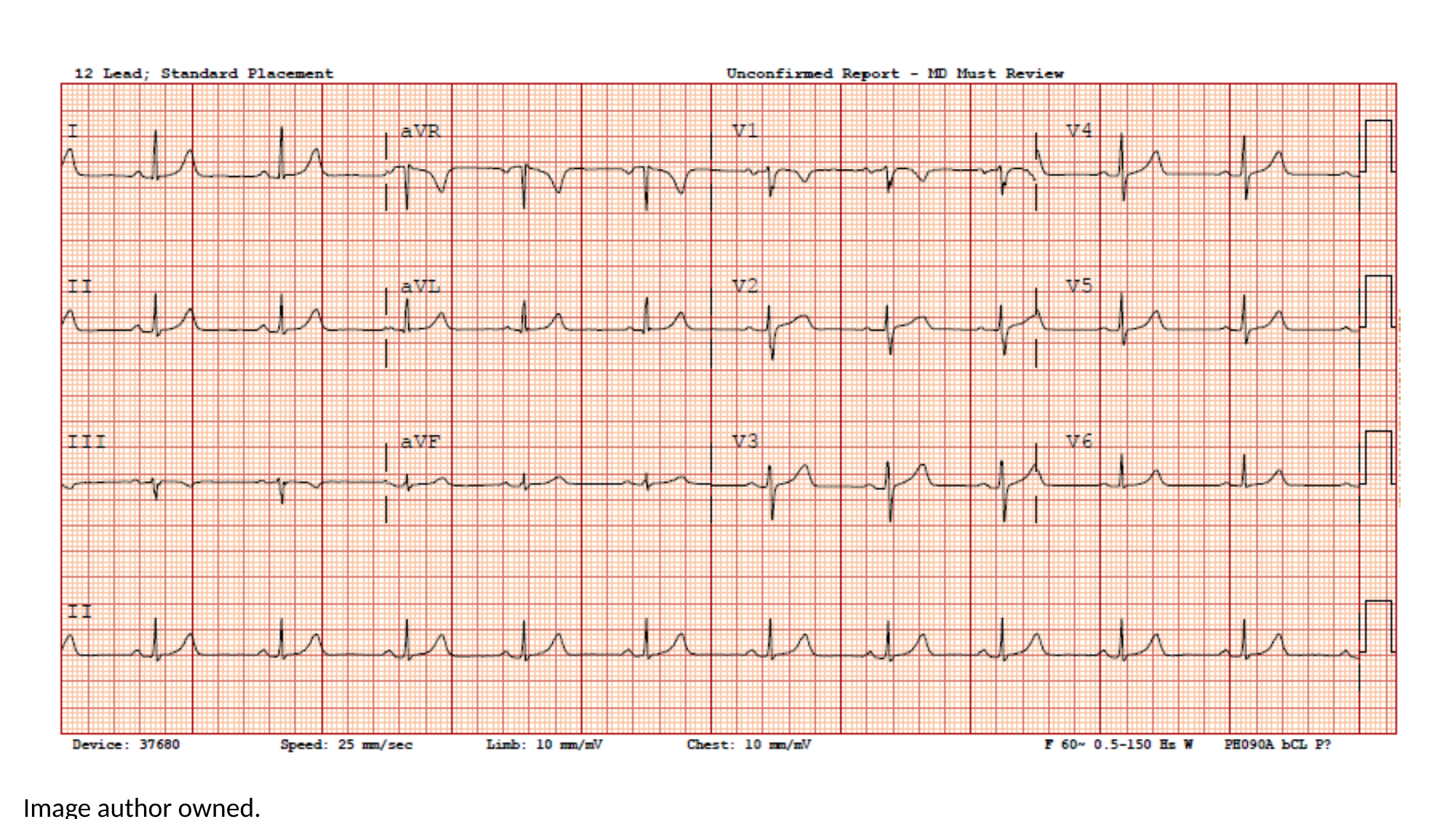

Image author owned.

## Slide 4
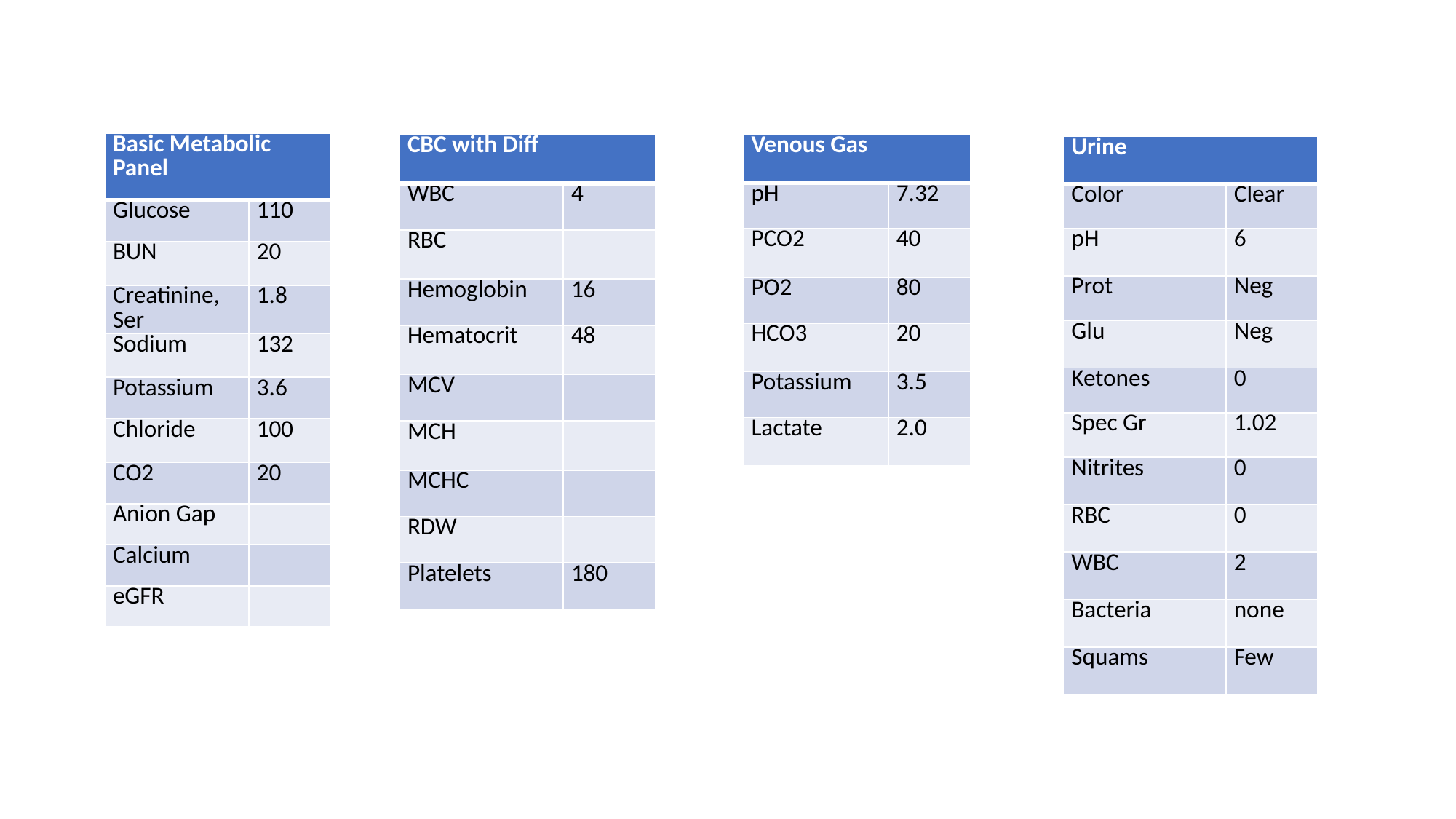

| Basic Metabolic Panel | |
| --- | --- |
| Glucose | 110 |
| BUN | 20 |
| Creatinine, Ser | 1.8 |
| Sodium | 132 |
| Potassium | 3.6 |
| Chloride | 100 |
| CO2 | 20 |
| Anion Gap | |
| Calcium | |
| eGFR | |
| CBC with Diff | |
| --- | --- |
| WBC | 4 |
| RBC | |
| Hemoglobin | 16 |
| Hematocrit | 48 |
| MCV | |
| MCH | |
| MCHC | |
| RDW | |
| Platelets | 180 |
| Venous Gas | |
| --- | --- |
| pH | 7.32 |
| PCO2 | 40 |
| PO2 | 80 |
| HCO3 | 20 |
| Potassium | 3.5 |
| Lactate | 2.0 |
| Urine | |
| --- | --- |
| Color | Clear |
| pH | 6 |
| Prot | Neg |
| Glu | Neg |
| Ketones | 0 |
| Spec Gr | 1.02 |
| Nitrites | 0 |
| RBC | 0 |
| WBC | 2 |
| Bacteria | none |
| Squams | Few |

## Slide 5
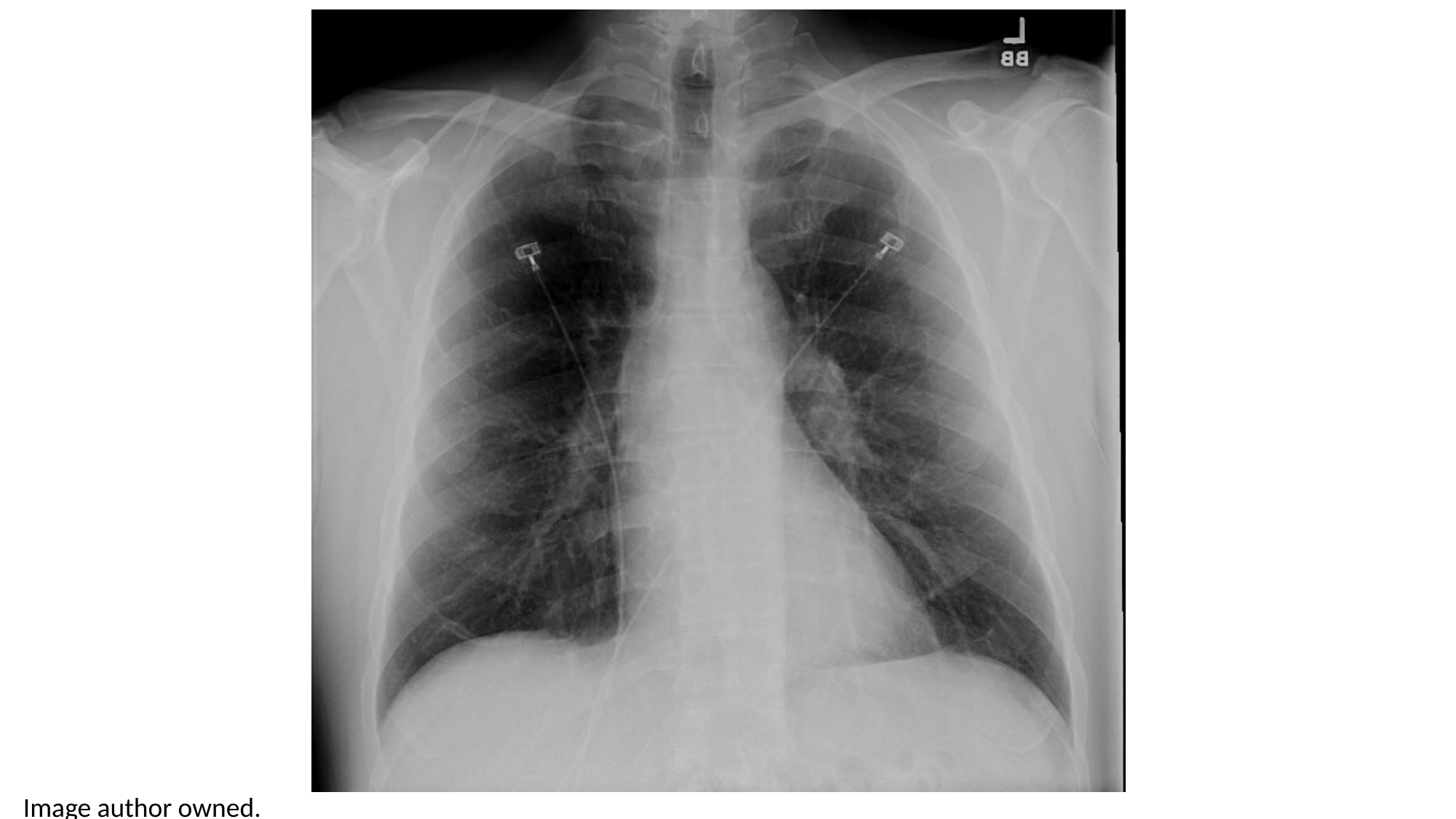

Image author owned.

## Slide 6
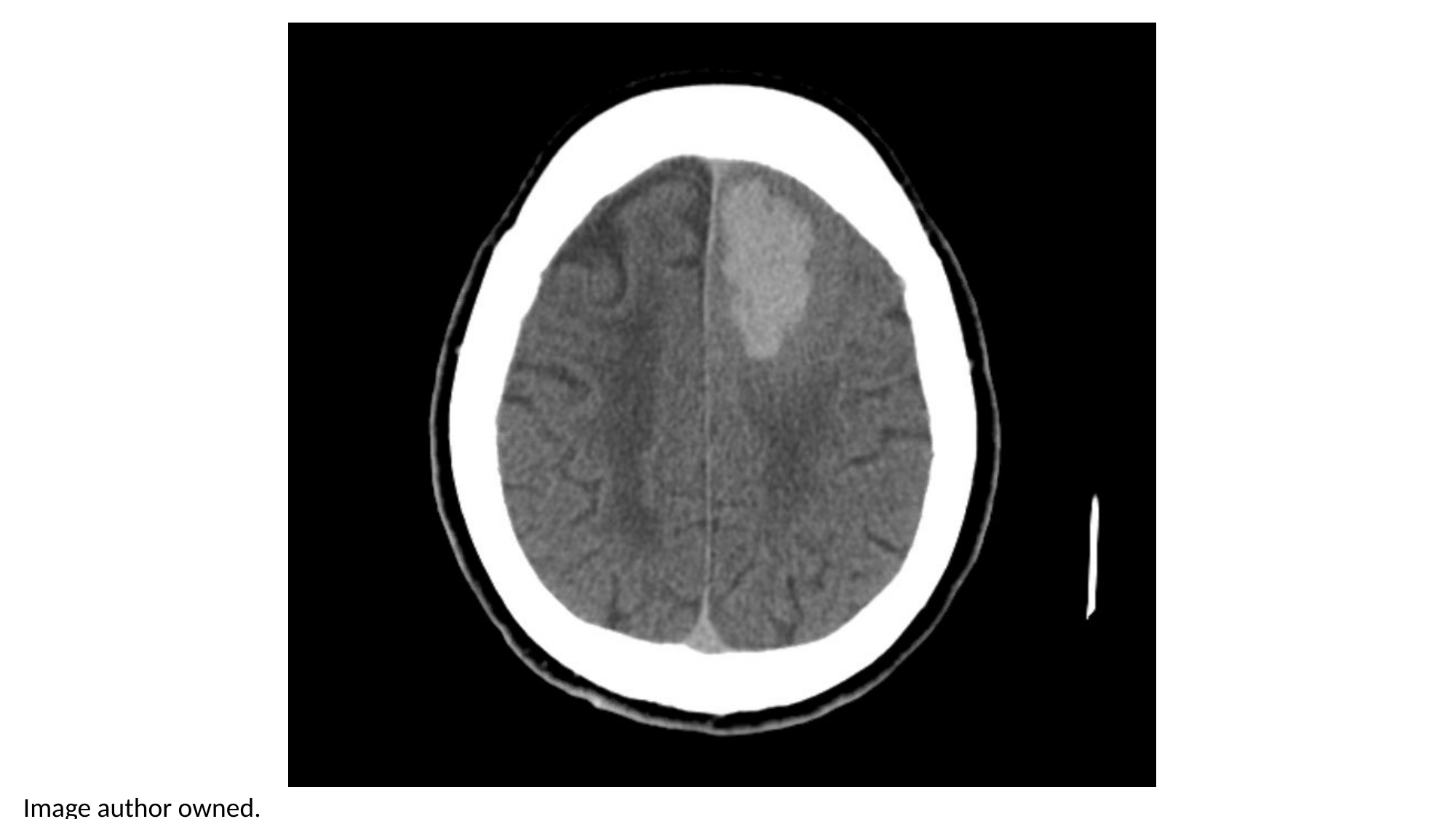

Image author owned.

## Slide 7
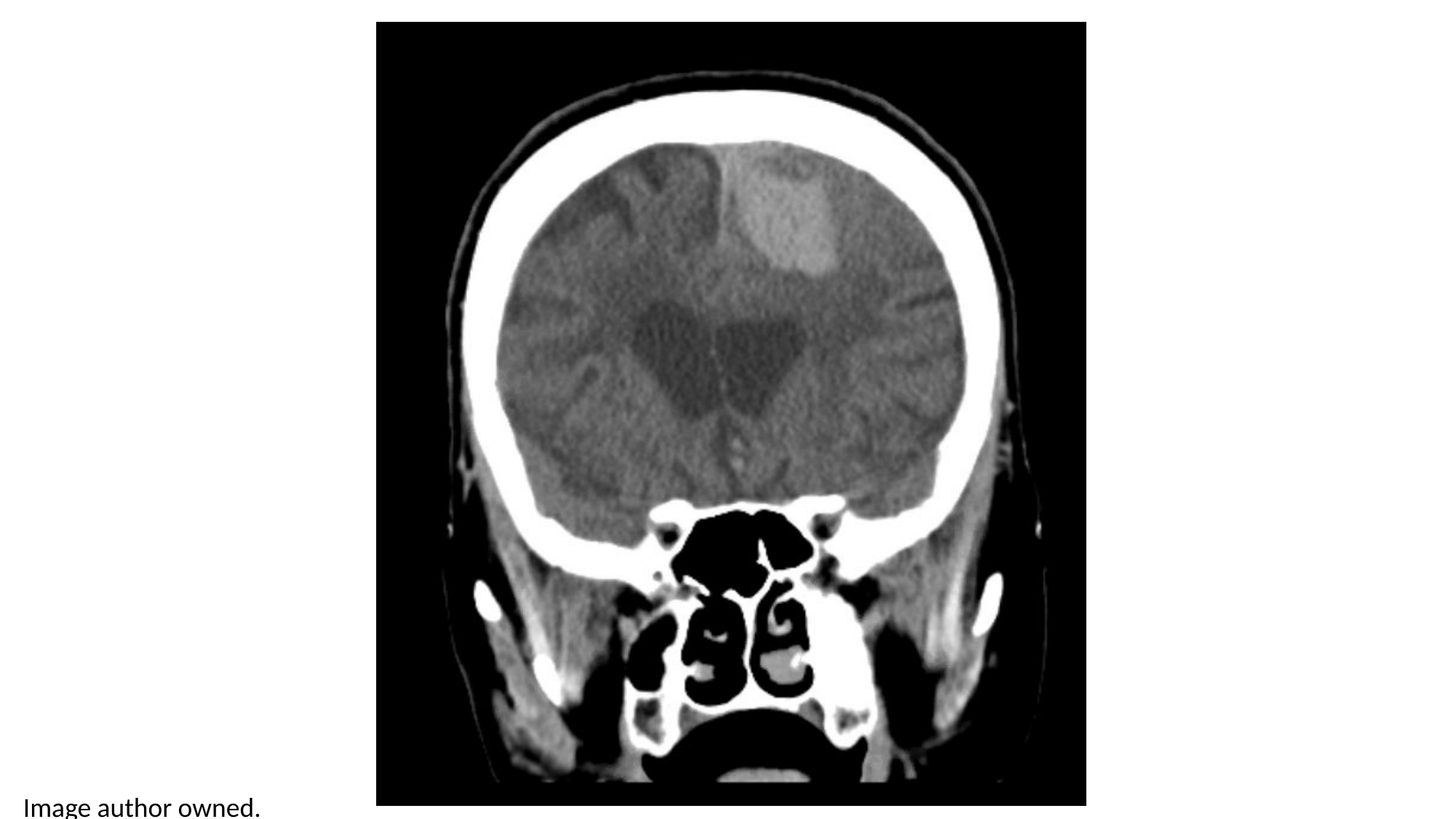

Image author owned.

## Slide 8
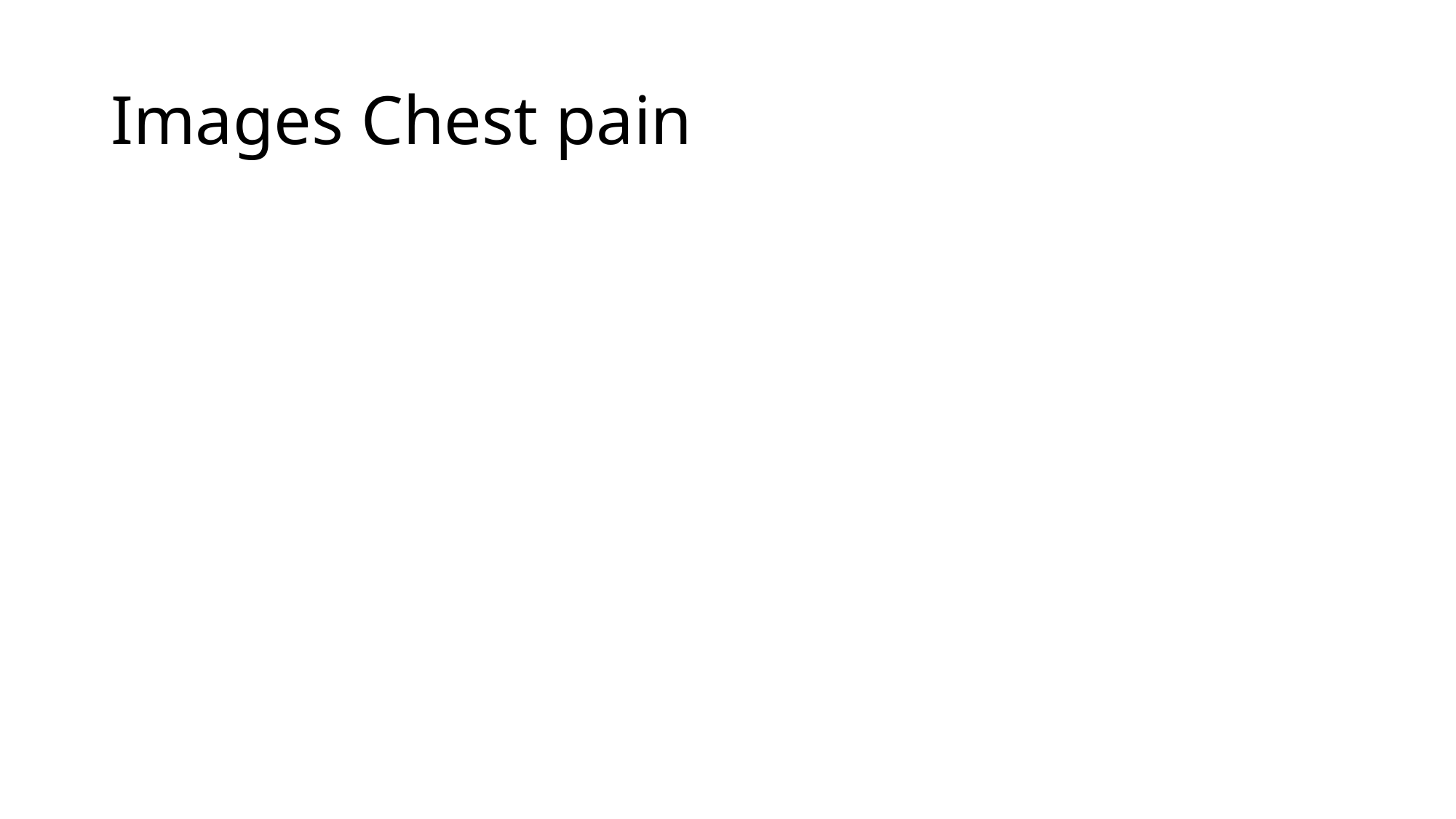

# Images Chest pain

## Slide 9
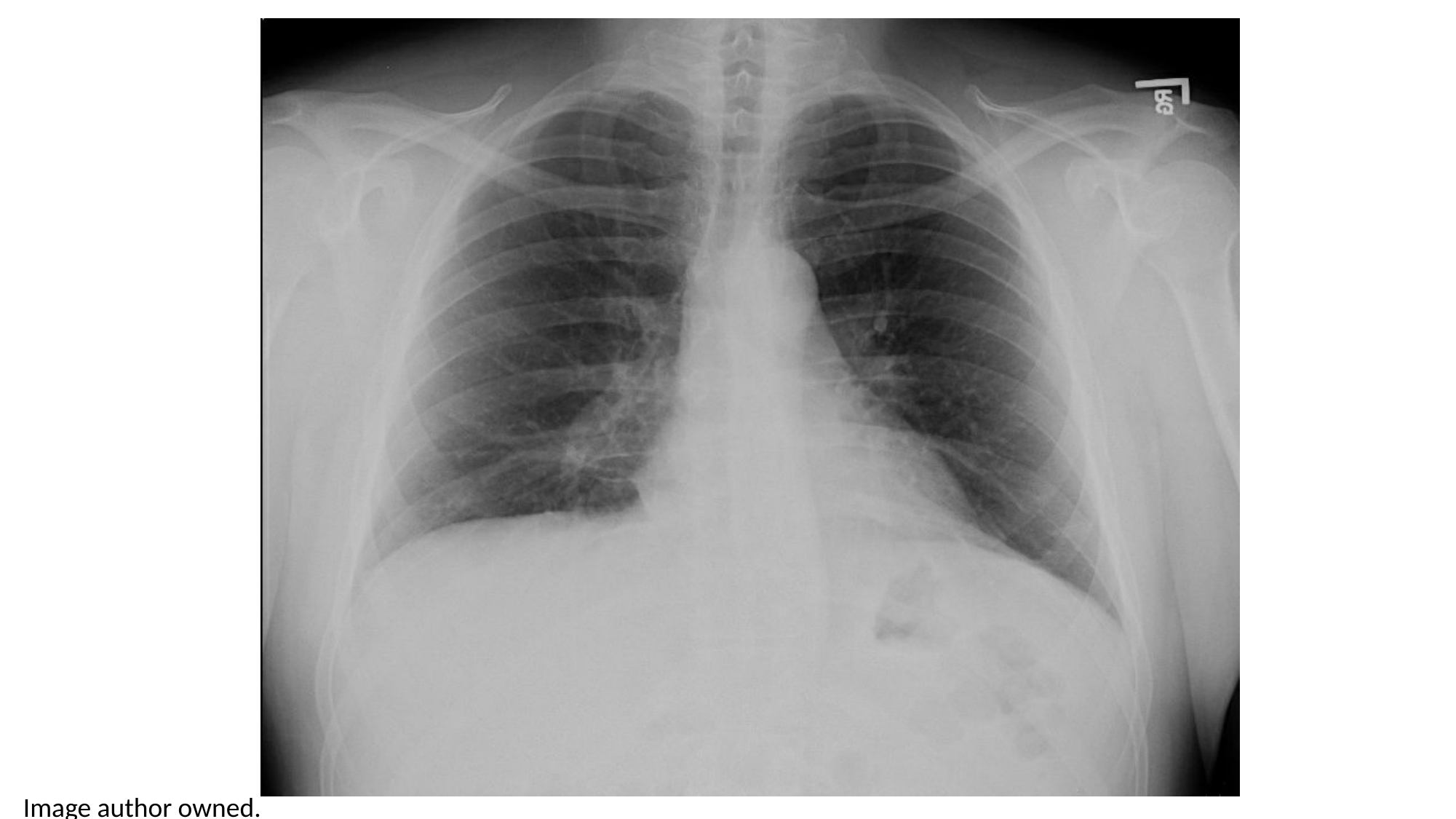

Image author owned.

## Slide 10
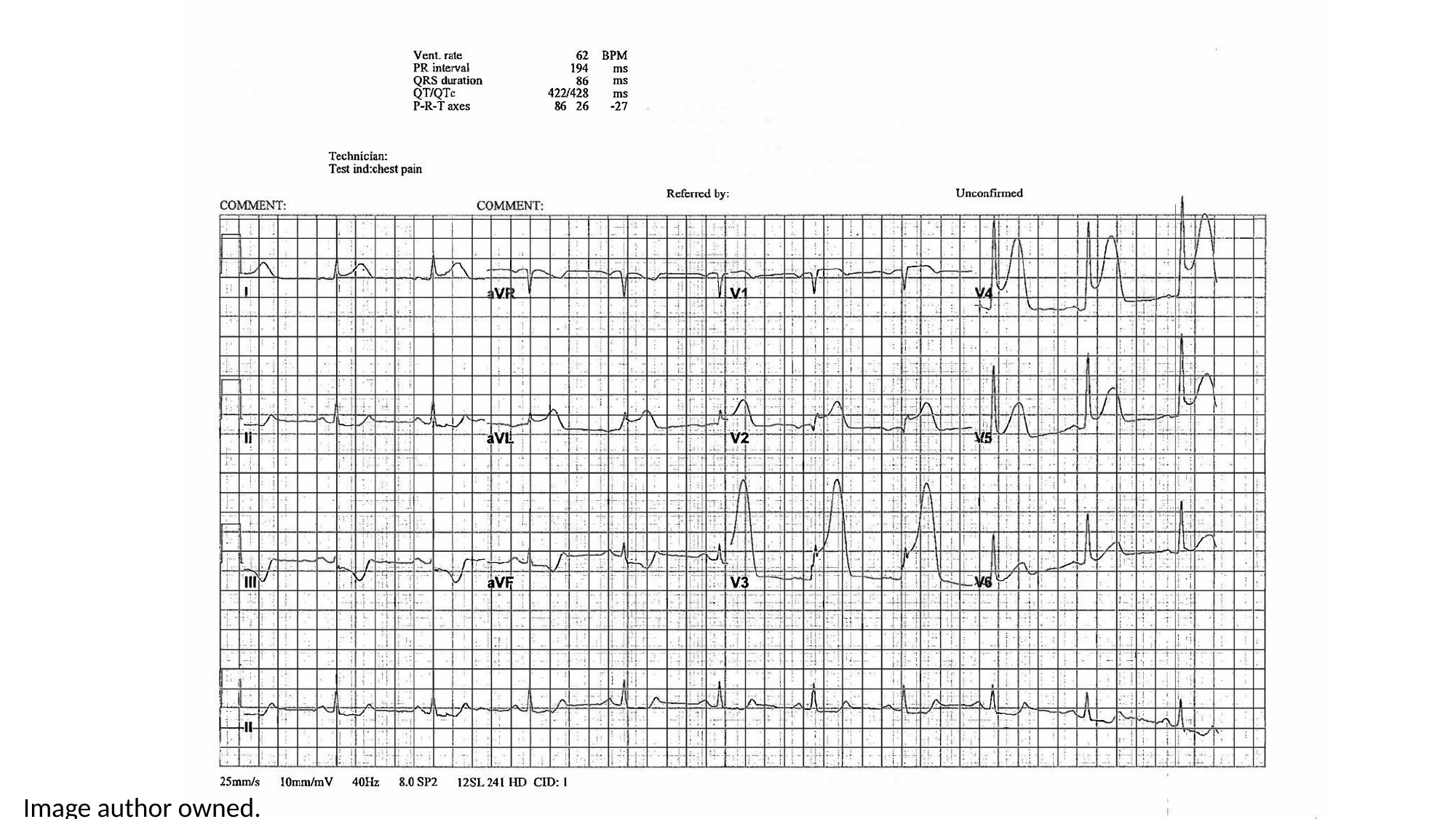

Image author owned.

## Slide 11
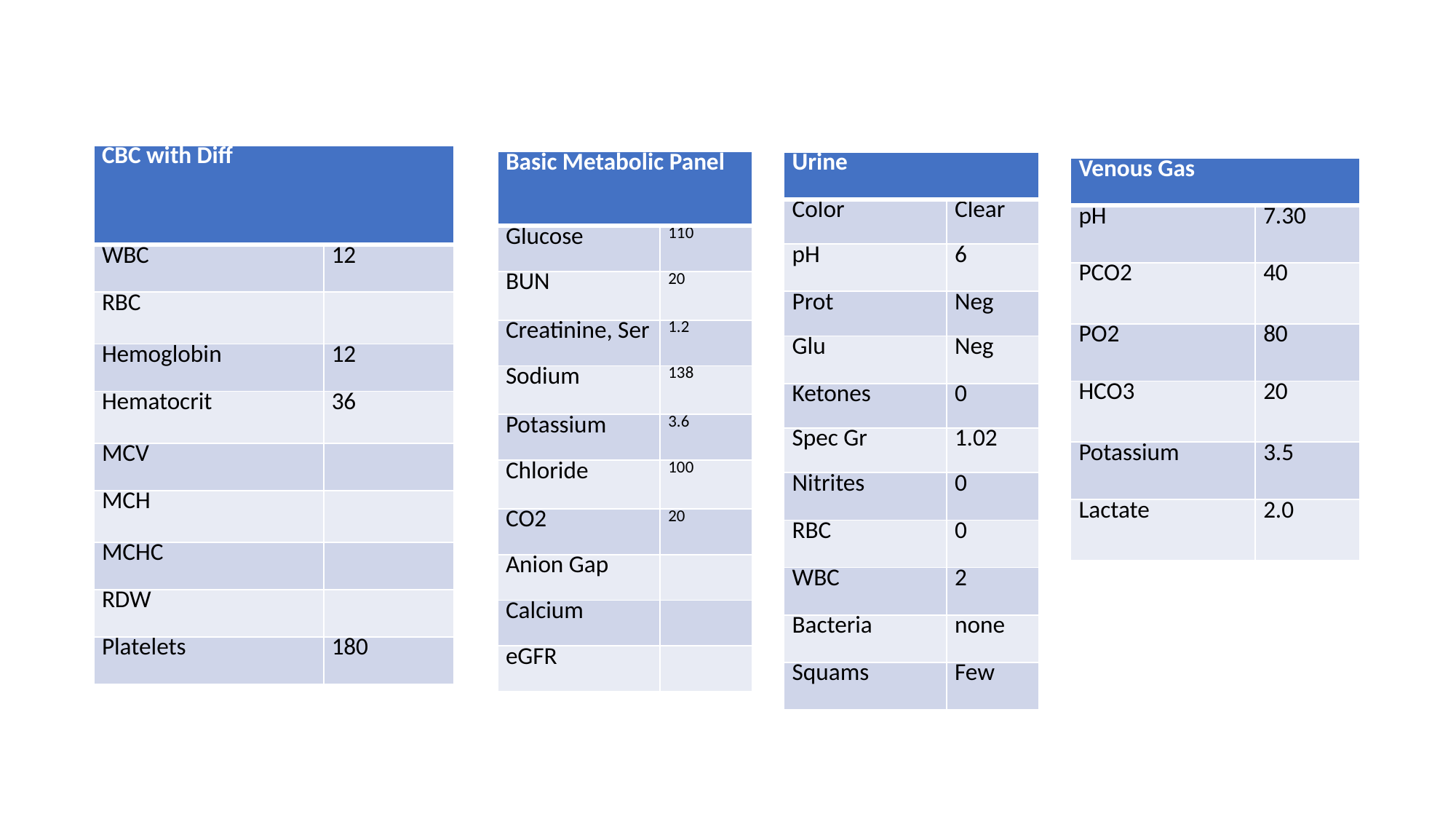

| CBC with Diff | |
| --- | --- |
| WBC | 12 |
| RBC | |
| Hemoglobin | 12 |
| Hematocrit | 36 |
| MCV | |
| MCH | |
| MCHC | |
| RDW | |
| Platelets | 180 |
| Basic Metabolic Panel | |
| --- | --- |
| Glucose | 110 |
| BUN | 20 |
| Creatinine, Ser | 1.2 |
| Sodium | 138 |
| Potassium | 3.6 |
| Chloride | 100 |
| CO2 | 20 |
| Anion Gap | |
| Calcium | |
| eGFR | |
| Urine | |
| --- | --- |
| Color | Clear |
| pH | 6 |
| Prot | Neg |
| Glu | Neg |
| Ketones | 0 |
| Spec Gr | 1.02 |
| Nitrites | 0 |
| RBC | 0 |
| WBC | 2 |
| Bacteria | none |
| Squams | Few |
| Venous Gas | |
| --- | --- |
| pH | 7.30 |
| PCO2 | 40 |
| PO2 | 80 |
| HCO3 | 20 |
| Potassium | 3.5 |
| Lactate | 2.0 |

## Slide 12
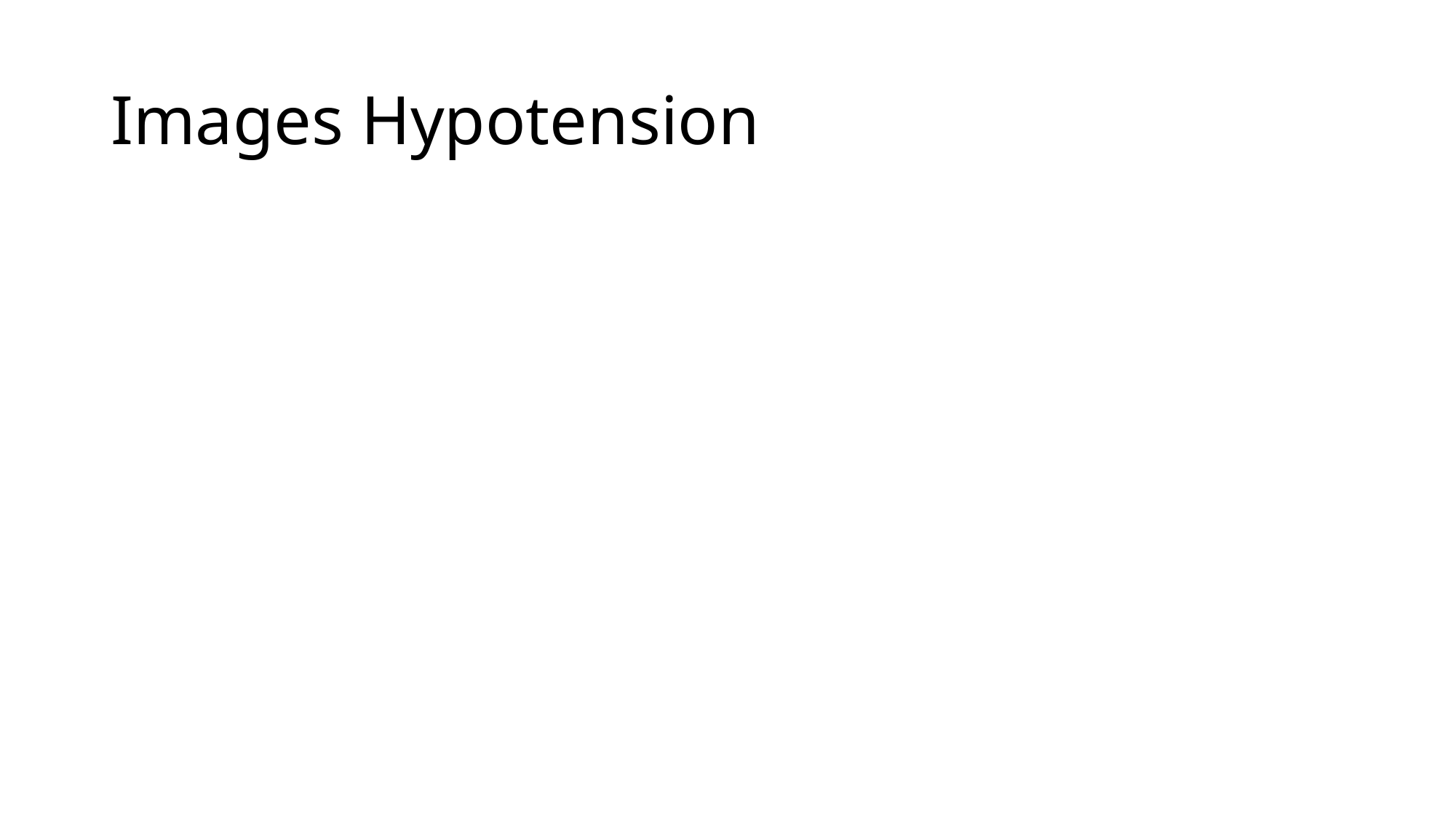

# Images Hypotension

## Slide 13
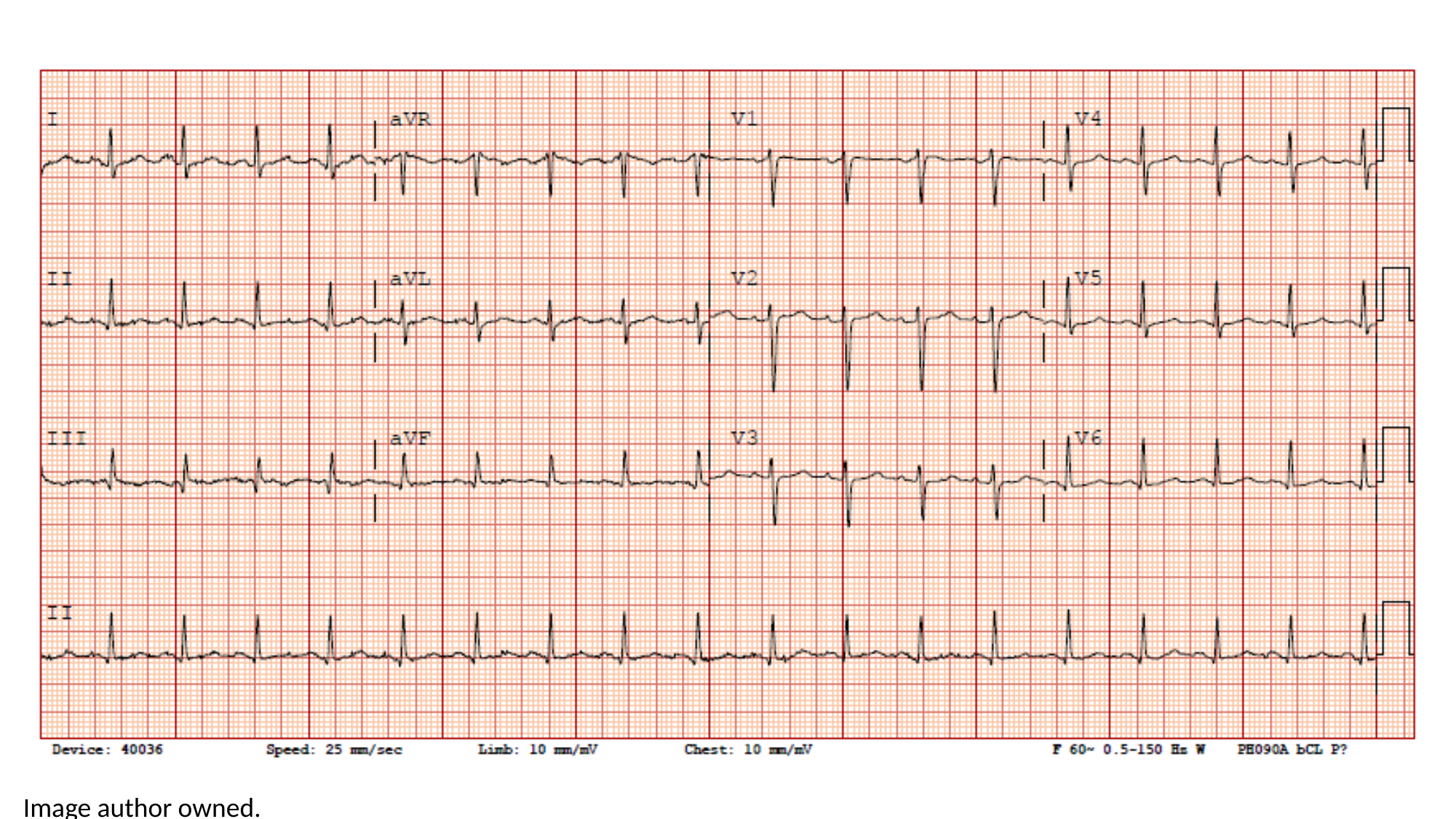

Image author owned.

## Slide 14
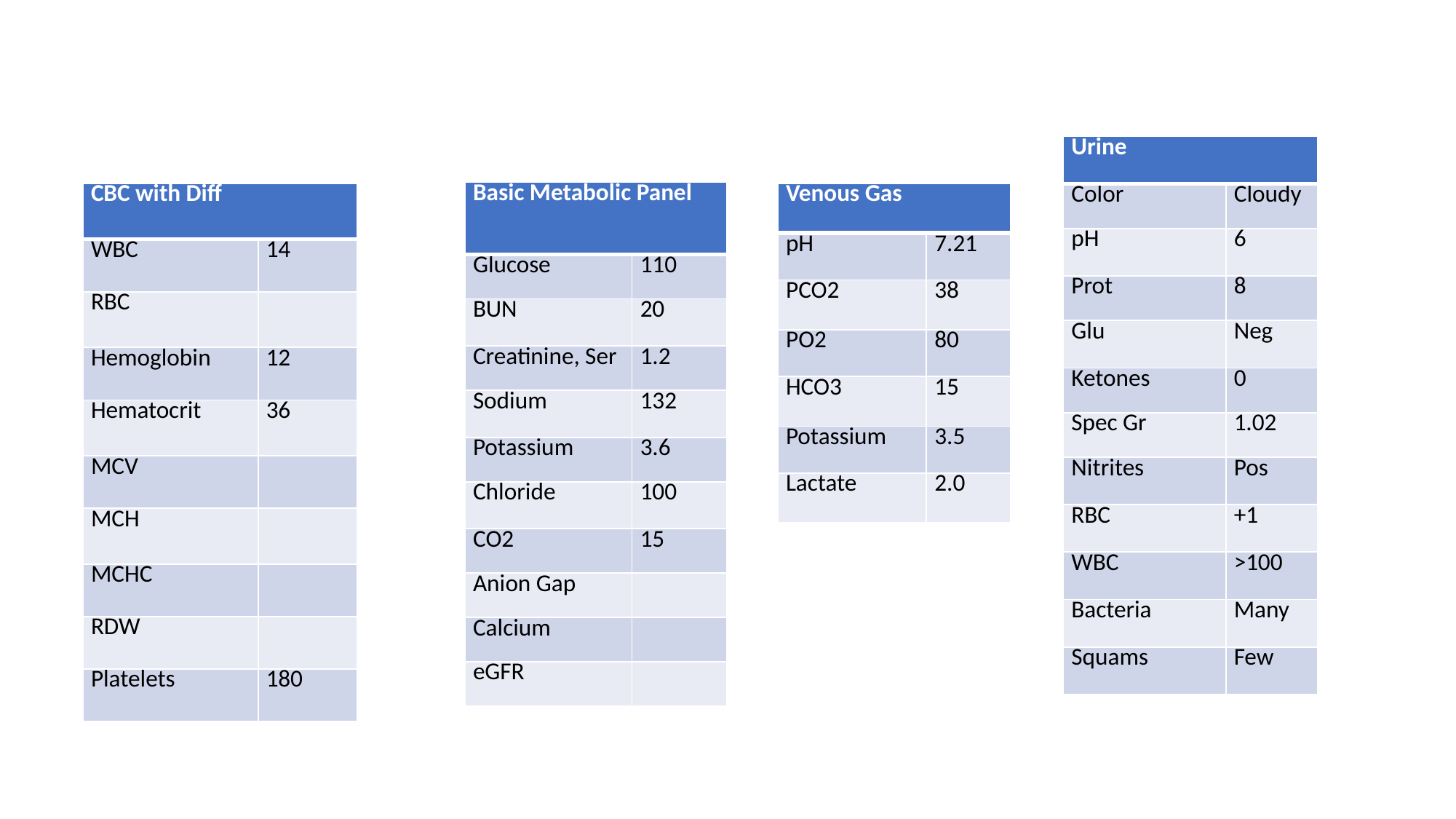

| Urine | |
| --- | --- |
| Color | Cloudy |
| pH | 6 |
| Prot | 8 |
| Glu | Neg |
| Ketones | 0 |
| Spec Gr | 1.02 |
| Nitrites | Pos |
| RBC | +1 |
| WBC | >100 |
| Bacteria | Many |
| Squams | Few |
| Basic Metabolic Panel | |
| --- | --- |
| Glucose | 110 |
| BUN | 20 |
| Creatinine, Ser | 1.2 |
| Sodium | 132 |
| Potassium | 3.6 |
| Chloride | 100 |
| CO2 | 15 |
| Anion Gap | |
| Calcium | |
| eGFR | |
| CBC with Diff | |
| --- | --- |
| WBC | 14 |
| RBC | |
| Hemoglobin | 12 |
| Hematocrit | 36 |
| MCV | |
| MCH | |
| MCHC | |
| RDW | |
| Platelets | 180 |
| Venous Gas | |
| --- | --- |
| pH | 7.21 |
| PCO2 | 38 |
| PO2 | 80 |
| HCO3 | 15 |
| Potassium | 3.5 |
| Lactate | 2.0 |

## Slide 15
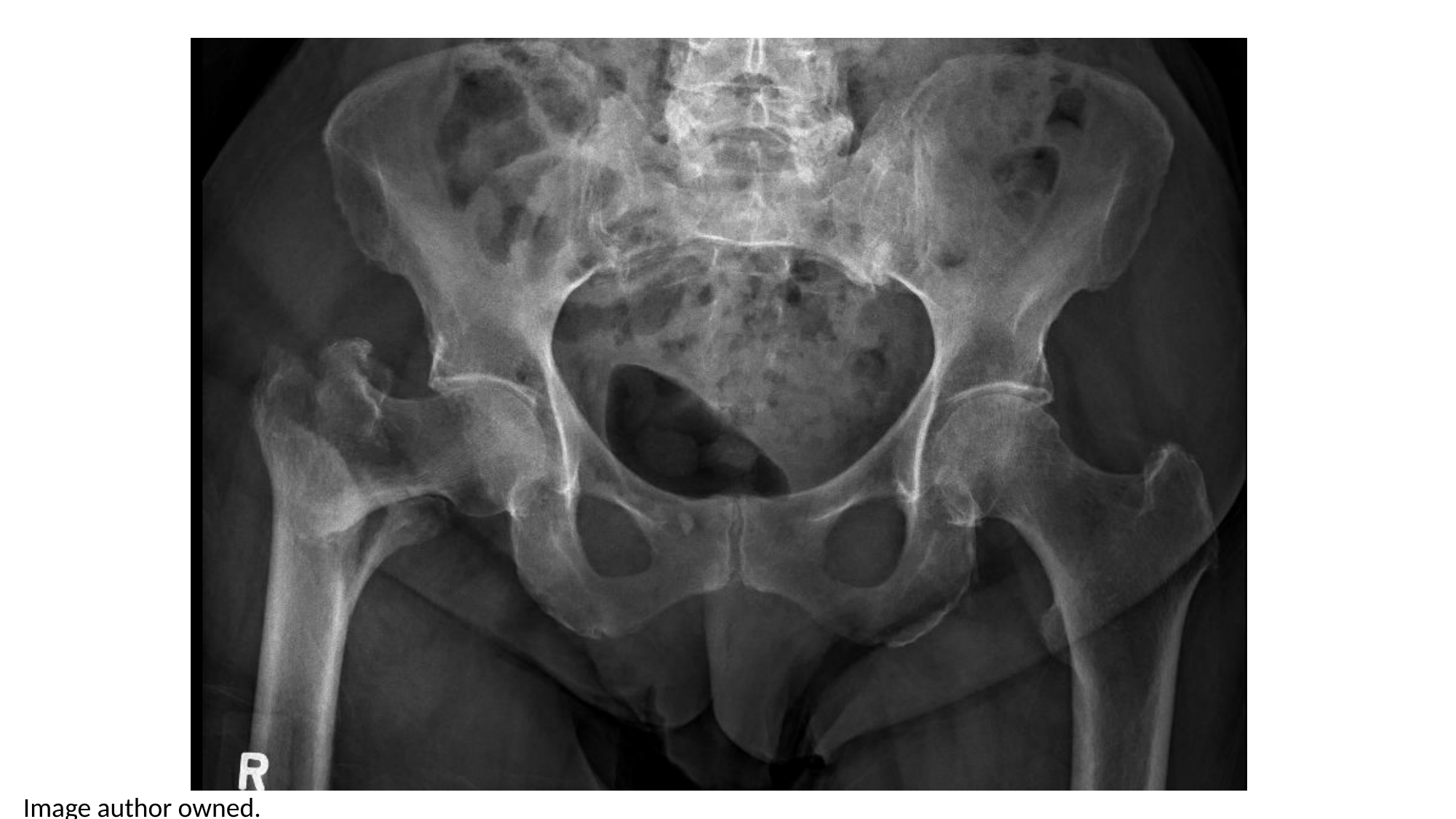

Image author owned.

## Slide 16
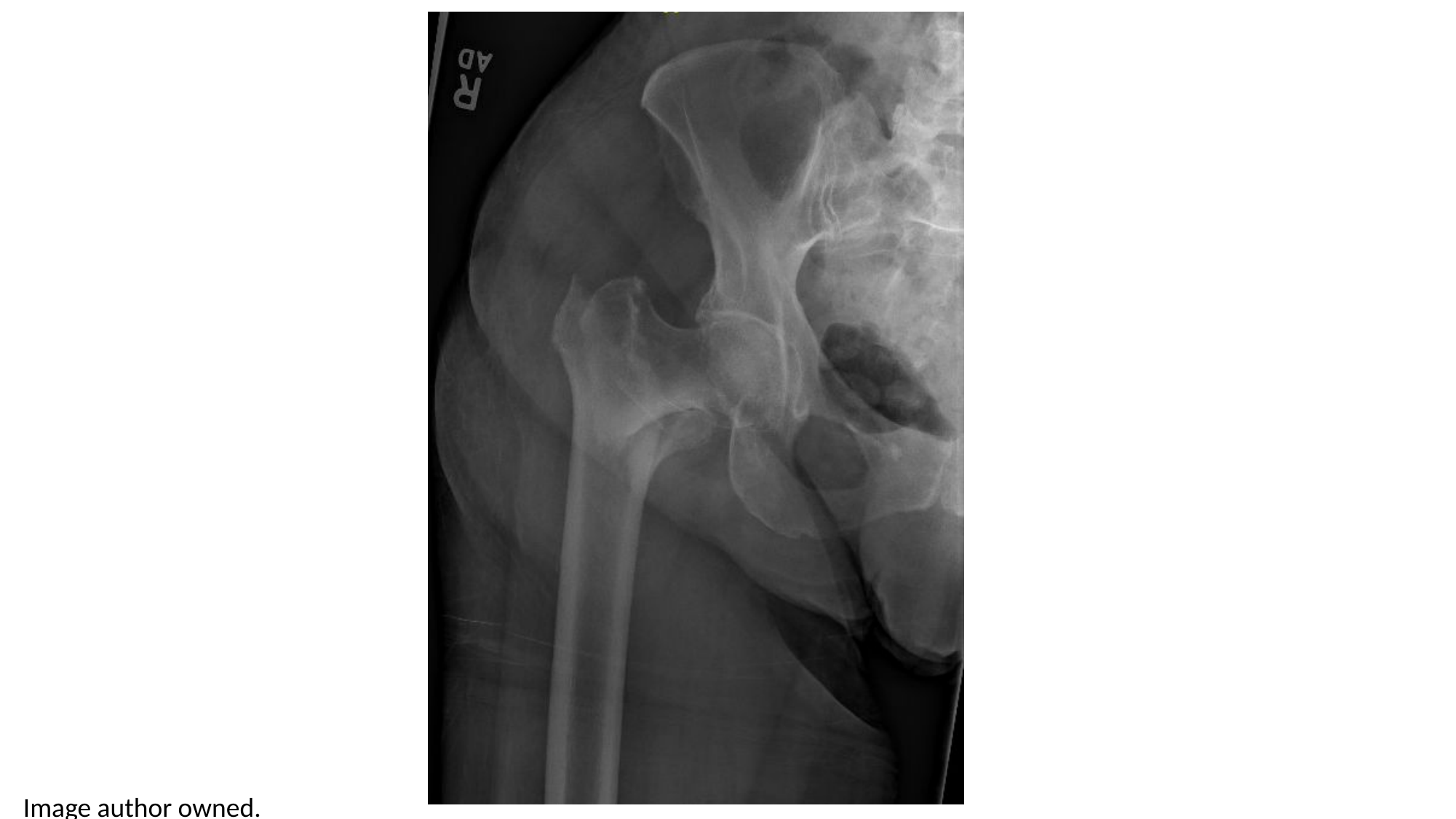

Image author owned.

## Slide 17
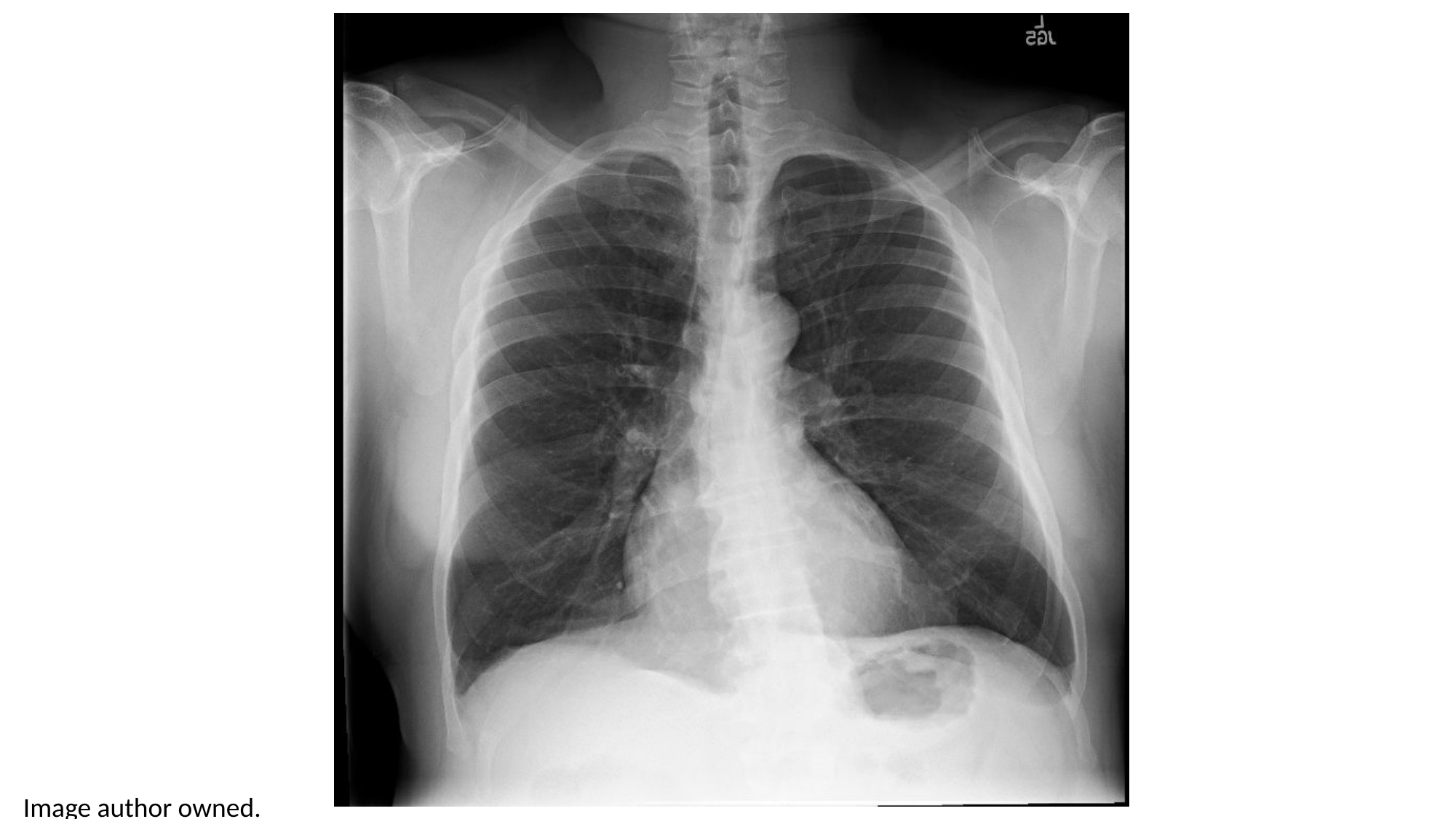

Image author owned.
